# Genome wide association study of incomplete hippocampal inversion in adolescents

**DOI:** 10.1371/journal.pone.0227355

**Published:** 2020-01-28

**Authors:** Claire Cury, Marzia Antonella Scelsi, Roberto Toro, Vincent Frouin, Eric Artiges, Antoine Grigis, Andreas Heinz, Hervé Lemaître, Jean-Luc Martinot, Jean-Baptiste Poline, Michael N. Smolka, Henrik Walter, Gunter Schumann, Andre Altmann, Olivier Colliot, the IMAGEN Consortium

**Affiliations:** 1 Centre for Medical Image Computing, Department of Medical Physics and Biomedical Engineering, University College London, London, England, United Kingdom; 2 Centre National de la Recherche Scientifique, Genes, Synapses and Cognition, URA, Institut Pasteur, Paris, France; 3 Human Genetics and Cognitive Functions, Institut Pasteur, Paris, France; 4 NeuroSpin, CEA, Université Paris-Saclay, Gif-sur-Yvette, France; 5 Inserm U 1000 “Neuroimaging & Psychiatry”, University Paris Sud, University Paris Descartes—Sorbonne Paris Cité, and Psychiatry Department 91G16, Orsay Hospital, France; 6 Charité –Universitätsmedizin Berlin, Department of Psychiatry and Psychotherapy, Campus Charité Mitte, Charitéplatz 1, Berlin, Germany; 7 Inserm U 1000 “Neuroimaging & Psychiatry”, Faculté de médecine, Université Paris-Sud, Le Kremlin-Bicêtre, and Université Paris Descartes, Sorbonne Paris Cité, Paris, France; 8 Inserm U 1000 “Neuroimaging & Psychiatry”, University Paris Sud, University Paris Descartes—Sorbonne Paris Cité, and Maison de Solenn, Paris, France; 9 McGill University, Faculty of Medicine, Montreal Neurological Institute and Hospital, McConnell Brain Imaging Center, Ludmer Centre for Neuroinformatics and Mental Health, Canada; 10 Department of Psychiatry and Neuroimaging Center, Technische Universität Dresden, Dresden, Germany; 11 Medical Research Council—Social, Genetic and Developmental Psychiatry Centre, Institute of Psychiatry, Psychology & Neuroscience, King’s College London, England, United Kingdom; 12 Institut du Cerveau et de la Moelle épinière, ICM, Paris, France; 13 Inserm, U 1127, Paris, France; 14 CNRS, UMR 7225, Paris, France; 15 Sorbonne Université, Paris, France; 16 Inria, Aramis project-team, Paris, France; Max-Planck-Institut fur Psychiatrie, GERMANY

## Abstract

Incomplete hippocampal inversion (IHI), also called hippocampal malrotation, is an atypical presentation of the hippocampus present in about 20% of healthy individuals. Here we conducted the first genome-wide association study (GWAS) in IHI to elucidate the genetic underpinnings that may contribute to the incomplete inversion during brain development. A total of 1381 subjects contributed to the discovery cohort obtained from the IMAGEN database. The incidence rate of IHI was 26.1%. Loci with P<1e-5 were followed up in a validation cohort comprising 161 subjects from the PING study. Summary statistics from the discovery cohort were used to compute IHI heritability as well as genetic correlations with other traits. A locus on 18q11.2 (rs9952569; OR = 1.999; Z = 5.502; P = 3.755e-8) showed a significant association with the presence of IHI. A functional annotation of the locus implicated genes *AQP4* and *KCTD1*. However, neither this locus nor the other 16 suggestive loci reached a significant p-value in the validation cohort. The *h*^*2*^ estimate was 0.54 (sd: 0.30) and was significant (Z = 1.8; P = 0.036). The top three genetic correlations of IHI were with traits representing either intelligence or education attainment and reached nominal P< = 0.013.

## Introduction

**Fig 1:**
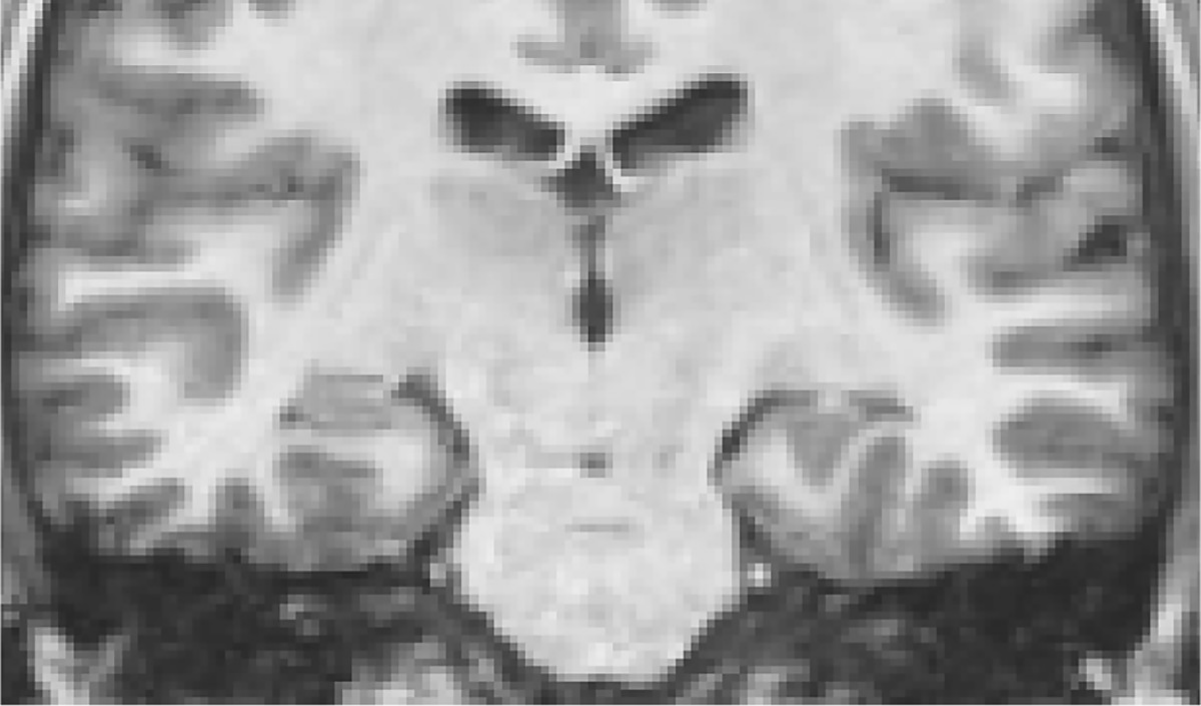
T1 weighted MRI in a coronal view. The left hippocampus (right side in the image) presents an incomplete hippocampal inversion (IHI). The right hippocampus (left side in the image) is an example of a normal or properly inverted hippocampus.

Human hippocampi are small structures, one in each temporal lobe that belongs to the brain’s limbic system and is known to be mainly involved in memory processes such as long term memorisation and spatial navigation [1]. The limbic system and the hippocampus influence the activity of the hypothalamic Pituitary Adrenocortical (HPA) axis, a major neuroendocrine mediator of stress, playing a role in emotional stress responses [2]. Thus, the hippocampus is implicated, with evidence of morphological changes, in a variety of neurological pathologies and psychiatric disorders, such as Alzheimer's disease where hippocampal atrophy increases with the pathology [3]; major depressive disorder where hippocampal volume can predict the response to antidepressants [4,5], is related to suicide attempts [6], and is linked to cortisol disruption (highlighting the implication of the hippocampus in the HPA axis) [7]; Schizophrenia, where patients have smaller hippocampi [8]; or temporal lobe epilepsy, the most frequent form of chronic focal epilepsy in adults, linked to hippocampal sclerosis [9]. Furthermore, during brain development, the growth of the left and the right hippocampi shows distinct responses to postnatal maternal stress [10]. Anatomically, there is a variation to the typical presentation of the hippocampi in normal subjects: the incomplete hippocampal inversion (IHI) also referred to as hippocampal malrotation (Fig 1). This anatomical variant has been initially observed in healthy subjects by [11] and then mostly observed in patients with epilepsy [12,13]. IHIs are mainly left-sided and characterized by a rounded or vertical shape, a medial positioning and a deep collateral sulcus [13–15] and are present in around 20% of the normal population [15]. It has been reported that IHI impacts the hippocampal volume: subjects with incomplete inversions appear to have smaller hippocampi [16], and more specifically, the hippocampal subfield CA1 seems to be related to the IHI severity [17]. Also it has been suggested that IHI might interfere with the quality of hippocampal segmentation for volumetric analysis [16,18], which may be clinically relevant, since the hippocampal volume can predict the response to antidepressant in patients without IHI [4,5]. Additionally, a sulcal morphometry analysis suggested that morphological changes associated with IHI are not confined to the hippocampus [15]; significant differences in cortical sulci located along the limbic system are shown between participants with and without complete inversion. Several studies suggest that IHI have their origin in developmental processes [19,20]. For example, [21] observed that during the rotational growth of the hemispheres, the major portion of the hippocampus is carried dorso-laterally and then ventrally to lie in the medial part of the temporal lobe. As the neocortex expands and evolves, the allocortex (the 3 layers cortex) is displaced inferiorly, medially and internally into the temporal horn. This rotational growth of the cortex implies an inversion of the hippocampus during normal development, which in some cases may remain incomplete. Following this hypothesis, [22] conducted a study using foetal MRI and found a correlation between the degree of in-folding and the number of gestational weeks. In a recent study [15] described detailed criteria to evaluate IHI, ultimately making the IHI evaluation more reproducible. In the same study, the introduced criteria had been applied to assess the IHI status of 2000 adolescents without neurological disorders. Results showed a prevalence of about 20% of IHI among this normal population. The majority of the IHI cases were left-sided (17% on left side). The lateral preference of left-sided over right-sided IHI may be rooted in the observation of asymmetrical hippocampus development in neonates with the right hippocampus developing faster than the left one [23]. In addition to these developmental observations, IHI has been reported to be associated with genetic changes. For instance, IHI was observed at higher prevalence in subjects with chromosome 22q11.2 microdeletion [24], which leads to DiGeorge syndrome.

Given that recent evidence implicates developmental processes in the aetiology of IHI and the observation that the structure and shape of subcortical structures, including the hippocampus, are under genetic control [25], we aimed at elucidating specific genetic variants contributing to IHI. To this end we conducted the first genome-wide association study on the genetics of incomplete hippocampal inversion.

## Methods

### Subjects

Subjects were investigated from two cohorts: IMAGEN [26] and PING [27]. The IMAGEN cohort comprises >2000 subjects collected at eight sites across Europe [26], and local ethics committee approved the study (see at the end of the paper for details and study [26]). At the time of baseline data collection and study inclusion all participants were 14 years of age. The second cohort was obtained from the Pediatric Imaging Neurocognition and Genetics (PING) Study database (http://www.chd.ucsd.edu/research/ping-study.html). PING was launched in 2009 by the National Institute on Drug Abuse (NIDA) and the Eunice Kennedy Shriver National Institute Of Child Health & Human Development (NICHD) as a 2-year project of the American Recovery and Reinvestment Act. The primary goal of PING has been to create a data resource of highly standardized and carefully curated magnetic resonance imaging (MRI) data, comprehensive genotyping data, and developmental and neuropsychological assessments for a large cohort of developing children aged 3 to 20 years. The scientific aim of the project is, by openly sharing these data, to amplify the power and productivity of investigations of healthy and disordered development in children, and to increase understanding of the origins of variation in neurobehavioral phenotypes. Access to the dataset was granted through a Federal Wide Assurance (FWA). For up-to-date information, see http://www.chd.ucsd.edu/research/ping-study.html and [27]. All methods were performed in accordance with relevant guidelines and regulations.

### Image data processing and IHI scoring

**Fig 2:**
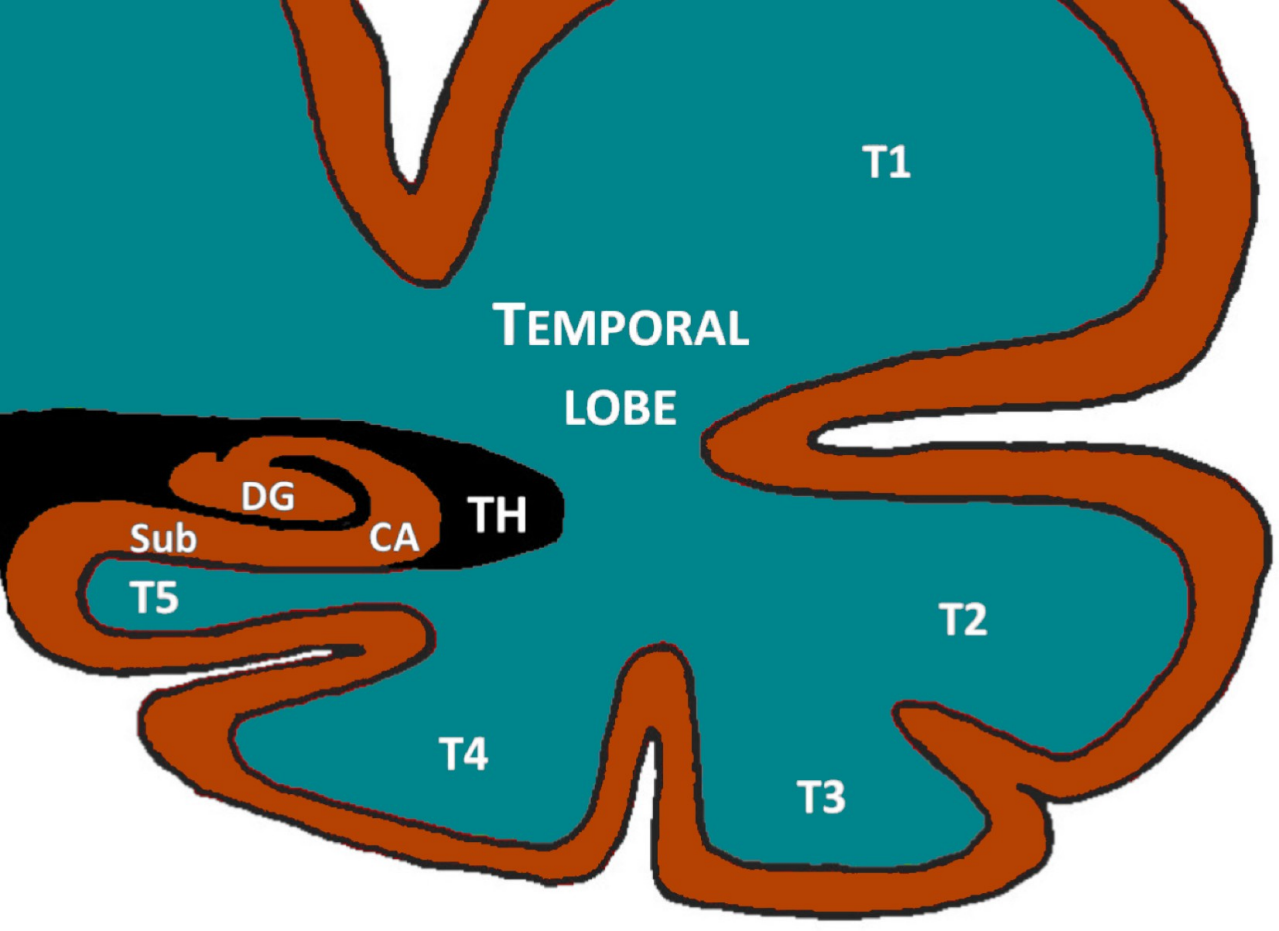
Hippocampal anatomy in coronal view. The hippocampus comprises the Dentate gyrus (DG), the cornus ammonis (CA) and the subiculum (sub). The temporal lobe is composed of five convolutions: T1, T2, T3, T4 and T5. The collateral sulcus divides T5 from T4 and the sulci of the fusiform gyrus separates T4 from T3. TH indicates the location of the temporal horn of the lateral ventricles.

**Fig 3:**
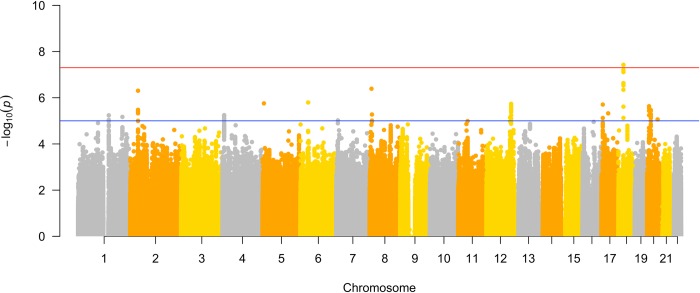
Manhattan plot. The y-axis depicts the -log10(p-value) of the association between SNP and presence of IHI assuming an additive model in the discovery cohort. The SNPs tested in the study are ordered along their chromosomal position on the x-axis. The red horizontal line donates genome wide significance at the Bonferroni threshold (P = 5e-8), while the blue horizontal line marks the threshold for suggestive association (P = 1e-5).

The procedure for scoring IHI [15], which has been previously described in detail and shown a good intra- and inter-reproducibility [15], was applied to the subjects used in this study (from IMAGEN and PING). Inter- and intra-rater variability were assessed in a previous publication [15]. This was studied on 42 participants from the discovery cohort using the kappa statistic. In all cases, intra- and inter-rater agreements were beyond substantial (κ≥0.64). Very strong agreements (κ≥0.8) were observed in the majority of comparisons (14/20). Rating on the validation cohort was conducted after by a single rater (CC), thus the rater was not blinded to whether subjects were from the discovery or validation cohort. In brief, the IHI score is composed of four different criteria: (1) assessing the roundness of the hippocampal body; (2) evaluating the verticality of the collateral sulcus which is located between the 4^th^ and the 5^th^ temporal lobe convolution (Fig 2); (3) the mediality of the hippocampal body; and (4) the depth of the fusiform gyrus, separating the 4th and the 3rd convolution of the temporal lobe (Fig 2). Each criterion is assessed from a coronal point of view after registering the subjects’ T1 weighted MRI into the standard MNI space using the FSL’s affine transformation FLIRT [28,29]. Evaluation was carried out using an inhouse Java interface (https://github.com/cclairec/viewerIHI_java). During scoring each criterion received a score between 0.0 and 2.0. The first three criteria have a step size of 0.5, the fourth criterion is binary (0 or 2), and the 5th criterion, assessed between 0 and 2, has a step size of 1.0. The sum of those criteria forms the overall IHI score ranging from 0.0 to 10.0. This is a semi-continuous score (with a step of 0.5), where an IHI score of 0.0 indicates the total absence of IHI, and a score of 8.0 represents a very pronounced presentation of IHI. In their previous study [15] established an optimal cut-off (at 3.75) of the overall IHI score to indicate presence or absence of IHI, by maximising the accuracy of the classification of a global criterion (blind to individual criteria or IHI scores), indicating if a given hippocampus presents or not an IHI (an intermediate score for partial IHI were present but not used in the estimation of the optimal cut-off): hippocampi without IHI correspond to IHI score < 4.0 and hippocampi with IHI correspond to IHI scores > = 4.

For this genetic study, the phenotype was IHI in either left or right hippocampus. To determine IHI, we applied the same cut-off of 4.0 for left and right hippocampi and used, for the IMAGEN cohort, the previously processed data from the IMAGEN study [15].

### SNP genotyping and pre-processing

IMAGEN subjects were genotyped from blood samples on 610-Quad SNP and 660-Quad SNP arrays from Illumina. Genetic data was available for 1,841 subjects. In a first round of quality control (QC) we performed subject-level QC by removing subjects with mismatching self-reported sex and genotype inferred sex (N = 10) or where more than 10% of SNPs were missing (N = 0). Next, we performed ancestry matching based on the HapMap3 data [30]. Population outliers were defined as subjects exhibiting more than five standard deviations distance from the CEU and TSI population in any of the first five principal components. Based on these criteria, 220 subjects were excluded from further analysis (S1 Fig). For the remaining subjects the genetic relationship matrix (GRM) was computed on common SNPs (minor allele frequency [MAF] >5%) after LD pruning using GCTA [31]. Another 18 subjects were removed due to relatedness (i.e., PIHAT > 0.05) leaving a total of 1593 subjects for the analysis. The raw genotyping data were prepared for imputation using a series of scripts (http://www.well.ox.ac.uk/~wrayner/tools/). Haplotype reference consortium (HRC) v1.1 [32] SNPs were imputed on the Sanger imputation server (https://imputation.sanger.ac.uk) using EAGLE2 [33] for pre-phasing and PBWT [34] for imputation. Data from the two different genotyping chips were imputed independently. Genotypes were hard called based on the maximal genotype posterior probability with a threshold of 0.9. That is, if none of the three genotypes reached a posterior probability of at least 0.9, then the SNP was set to missing in the corresponding subject. Finally, an additional round of QC was conducted on SNP level based on imputation quality (INFO score > 0.3), missingness (< 5%), minor allele frequency (MAF>1%) and deviation from Hardy-Weinberg-Equilibrium (p<1e-6) leaving 6,742,645 SNPs across the autosomes for the association analysis.

PING subjects were genotyped from saliva samples on Human660W-Quad arrays from Illumina. After QC, genetic data for 1,391 participants was suitable for analysis. Individual SNPs of the PING dataset were accessed through the PING data portal (ping-dataportal.ucsd.edu). Ancestry and admixture proportions in the PING participants were based on the ADMIXTURE software [35] and downloaded through the data portal (for details see [27]). We restricted the validation cohort to participants of at least 12 years of age and of European ancestry (minimum 90% European ancestry as per ADMIXTURE; N = 197).

### Genome wide association study

The genome wide association study was carried out with Plink v1.9 [36] assuming an additive genetic model and computing for every SNP a logistic regression while correcting for sex, age at imaging (in days) and five principal components for population structure. Phenotype or covariate information was missing for 212 participants. Thus, the discovery GWAS comprised 1,381 unrelated subjects. The genome-wide statistical significance threshold was set to the standard threshold of p<5e-8 and regional association plots were generated with LocusZoom [37]. SNPs exceeding the threshold for suggestive association with IHI (p<1e-5) were followed up in an independent cohort of adolescents (PING). In case the top SNP was not genotyped in PING, LDlink [38] (https://analysistools.nci.nih.gov/LDlink/) was used to identify a proxy in LD (r2) within +/- 50kb of the top SNP’s location. Association with single SNPs was tested in R using the glm function; the logistic model was corrected for age and sex.

### Functional annotation of GWA summary statistics

The GWA summary statistics were annotated using the web-based version of the FUnctional Mapping and Annotation (FUMA) tool [39] (http://fuma.ctglab.nl/). In order to elucidate the functional consequences of genetic risk loci, FUMA approaches the mapping in two separate steps: first, lead SNPs are identified and mapped to relevant genes on the basis of strand proximity, expression quantitative trait loci (eQTL) and chromatin interaction; second, the reprioritized genes returned by the first step are annotated with respect to expression levels and overrepresentation in differentially expressed gene sets among a wide range of human tissues.

For the purposes of this study, SNP-to-gene mapping was performed according to the following parameters: SNPs with p<5e-8 were identified as lead SNPs, and genomic risk loci were constructed by including SNPs in linkage disequilibrium with independent lead SNPs (LD r^2^>0.6 in the 1000 Genomes Phase 3 EUR panel) and with a minimum MAF of 1%. Positional mapping was performed by linking lead SNPs to genes in a 50kb window. Mapping based on eQTL was performed by using only SNP-gene pairs significant at FDR<0.05 in all tissues/cell types from 4 data repositories (GTEx [40], the Westra blood eQTL dataset [41], the BIOS QTL browser [42] and BRAINEAC [43]); the available data only covers *cis*-eQTLs with up to 1 Mb distance between SNP and gene. Chromatin interaction mapping was also performed to take into account potential long-range interactions between risk loci and genes due to chromatin folding. We based mapping on interactions significant at FDR<1e-6 in 14 tissue types and seven cell lines from [44]. We also based mapping on tissue/cell type specific enhancer or promoter regions annotated in 111 epigenomes from the Roadmap Epigenomics Project [45]. The Major Histocompatibility Complex (MHC) was excluded from annotations, and mapping to all functional gene classes (protein-coding, non-coding RNA, long intergenic ncRNA, processed transcripts, pseudogenes) was enabled.

After mapping lead SNPs to relevant genes, we performed annotation of the prioritized genes in biological context, mainly with respect to tissue-specific expression levels. Average expression levels (log_2_ Read Per Kilobase per Million (RPKM+1)) of protein-coding genes in 53 tissues from GTEx v6 were visualized through heat maps, allowing for comparison of expression level across genes and tissue types. Candidate genes were tested for overrepresentation in sets of differentially expressed genes (DEG), as well as sets of genes up- and down-regulated, across 53 specific tissue types from GTEx v6 using hypergeometric tests. The same gene-set enrichment analysis strategy was applied to test for overrepresentation of biological functions among the prioritized genes, using gene sets from the Molecular Signatures Database version 5.2 [46], WikiPathways [47] and the GWAS catalog [48], and applying the Benjamini-Hochberg multiple testing correction procedure.

### Heritability analysis and genetic correlation

We used LD score regression [49] in order to estimate IHI heritability from the GWAS summary statistics data. Next, we computed partitioned heritability estimates using the LD score method described in [50] and [51]. Heritability estimates were partitioned into 53 overlapping functional categories, derived from 24 main annotations, from [50]. Stratified LD score regression was also used to test for heritability enrichment in genes specifically expressed in a number of tissues of cell types. For this analysis, we used the specifically expressed gene lists compiled by [51] for the following datasets: expression levels from RNA-seq experiments in the 53 GTEx tissues and cell types, as well as only the 13 GTEx brain regions; the Cahoy dataset, comprising microarray expression data from three cell types (astrocyte, neuron, oligodendrocyte) in the mouse brain [52]; the Franke dataset, comprising microarray expression data in 152 tissues and cell types from human, mouse and rat [53]; and the Immunological Genome Project Consortium dataset, comprising microarray expression data for 292 immune cell types in the mouse [54]. Enrichment p-values were corrected for multiple comparisons using the Benjamini-Hochberg procedure.

Given the reported higher prevalence of IHI in patients with epilepsy, we used LD score regression to compute the genetic correlation [55] between IHI and epilepsy susceptibility based on a recent GWAS [56]. Finally, we conducted an exploratory analysis of genetic correlation between IHI and traits from 832 GWASs using the LD hub [57] (http://ldsc.broadinstitute.org/).

### Gene expression in the developing human brain

In order to explore the transcription pattern of two candidate genes, we downloaded their expression values from BrainSpan through the web interface (http://www.brainspan.org/rnaseq/search/index.html). The data comprises post-mortem gene expression data of 42 subjects at ages spanning from prenatal development (eight post conception weeks) till adulthood (40 years). Brains were sampled across 26 brain structures. Gene expression was measured using RNA-sequencing and expression levels for each gene were provided as reads per kilobase of exon model per million mapped reads (RPKM). We analysed this data using a linear mixed effects model implemented in the lme4 package in R. In these analyses, the gene expression level was the target variable, subject ID and structure ID were random effects, while an indicator variable for age less than 25 weeks post conception was the fixed effect. We tested for the significant effect of age<25 post conception weeks (pcw) on gene expression. This threshold was selected based on the estimated occurrence of hippocampal inversion between pcw 20 and 30 [20].

## Results

### Subjects: Cohort statistics

In IMAGEN 1381 subjects had genotyping and all phenotype and confounding information available. Incidence rate of IHI was 26.1%. In PING, for the 197 European subjects aged 12 years or older, we could successfully access and score 161 T1 weighted MR images for analysis, and IHI incidence rate was 23.6%; both at the 4.0 cutoff. There was a higher incidence rate of IHI in the left hemisphere in both cohorts. Summary statistics for both cohorts can be found in Table 1.

**Table 1:** Cohort summary statistics of participants used for the genetic analyses.

Cohort	IMAGEN	PING
Participants (N)	1381	161
Female (%)	687 (49.7)	78 (48.4%)
Age (SD)	14.5 (0.41)	16.06 (2.54)
IHI (%)	360 (26.1)	38 (23.6)
Left/Right/Bilateral	251/46/63	24/7/7

### Genome wide association study and functional annotation

**Fig 4:**
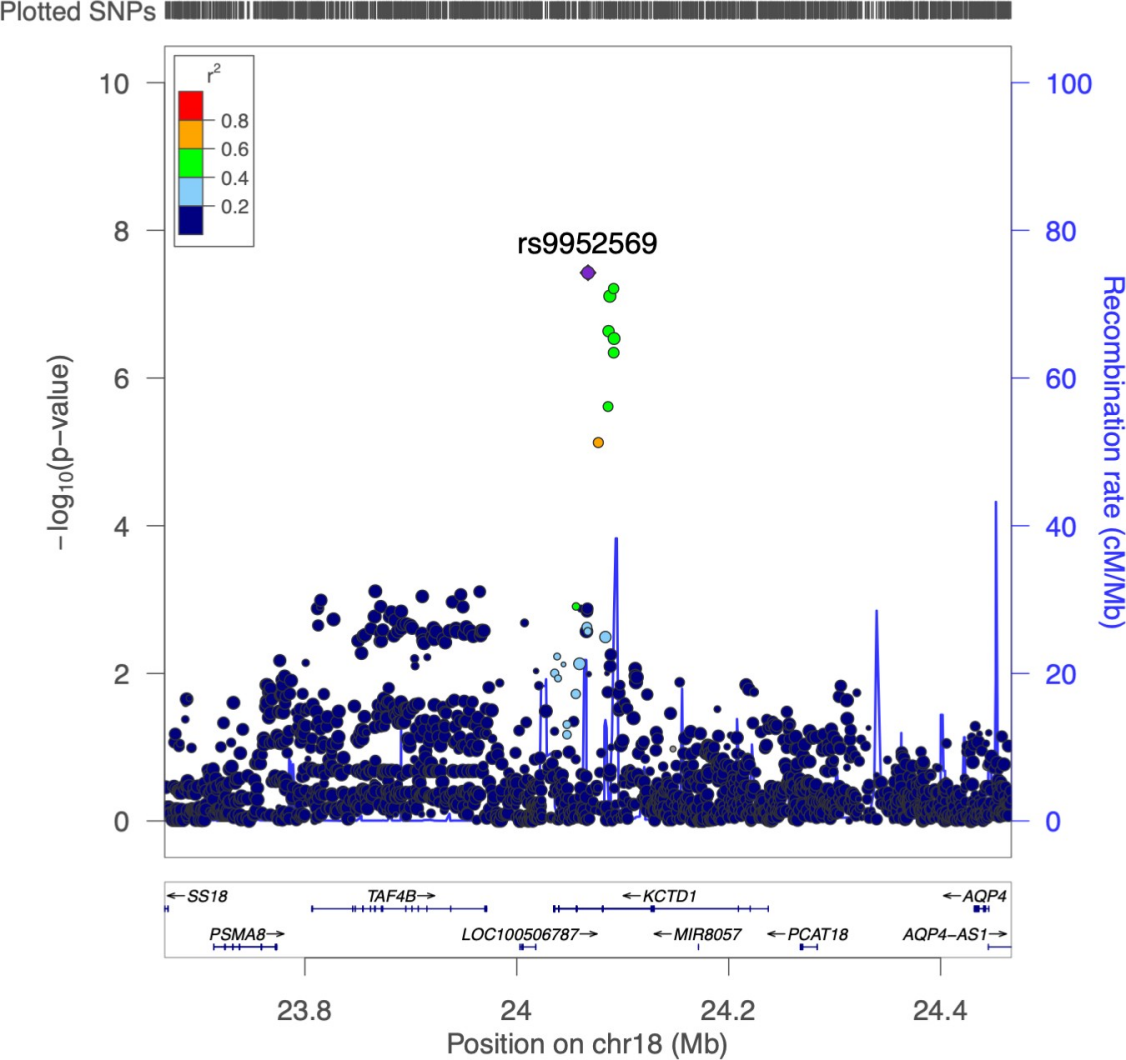
Regional association plot for rs9952569.

We tested each of the 6.7mio SNPs for an association with the presence of IHI. In the discovery dataset comprising subjects from the IMAGEN study, 17 loci passed the threshold for suggestive association (Fig 3; Table 2). One locus on chromosome 18 reached genome-wide significance (top SNP: rs9952569; OR = 1.999; Z = 5.502; P = 3.755e-8; Fig 4). There was no inflation in p-values (λ = 1.017; S2 Fig). The top SNP shows a consistently strong association with the continuous IHI score (i.e., not applying the cutoff at 4.0) and the global criterion (C0), but misses the genome-wide significance threshold in both cases (S3 Fig; beta = 0.5021, Z = 5.129, P = 3.332e-07 for the continuous score and beta = 0.2542, Z = 5.299, P = 1.354e-07 for C0). Functional annotation of the GWAS result was carried out using FUMA and linked the significant locus to six genes: *AQP4*, *AQP4-AS*, *CIAPIN1P*, *KCTD1*, *RNU6-1289P*, and *U3* (S4 Fig. left). In fact the top associated SNP is located in an intron of *KCTD1*. Brain gene expression based on GTEx shows high and brain-specific expression for *AQP4* and moderate to high expression levels for KCTD1 (S4 Fig, right).

**Table 2:** Suggestive loci from discovery GWAS and results in validation cohort (below). Left side: The table displays the leads SNPs in the discovery cohort (IMAGEN) with p<1e-5 along with their chromosome, base pair (BP), alleles (A1, A2), subjects (N), odds ratio (OR), statistics (STAT) and P-value. Right side: The second table depicts the p-values for the SNPs (VALSNP) in the validation cohort (PING), along with their base pair, correlation to the discovery SNP (R2), alleles (A1, A2), OR and P-value.

	Discovery									Validation							
CHR	SNP	BP	A1	A2	NMISS	OR	STAT	P	CHR	VALSNP	BP	R2	A1	A2	OR	STAT	P
1	rs79030318	147233364	A	G	1354	1.773	4.534	5.79E-06	1	rs12061877	147264252	0.755	C	T	1.5584465	1.0434	0.2331419
1	rs3009985	212191991	G	A	1369	1.513	4.501	6.77E-06	1	rs3738449	212240063	0.899	A	G	0.8060804	-0.7252	0.4743359
2	rs10490445	37617484	G	T	1379	1.812	5.027	4.97E-07	2	rs10490445	37617484	1	G	T	1.5846445	1.0391	0.1837016
4	rs6852934	7760243	A	G	1379	1.948	4.538	5.68E-06	4	rs2285769	7761286	1	A	G	1.4751031	0.5908	0.3685725
5	rs77126180	6478922	A	G	1337	3.31	4.78	1.75E-06	5	rs272456	6484012	0.042	A	G	0.7735566	-0.2829	0.3407685
6	rs35806781	37081139	A	G	1344	4.889	4.798	1.60E-06	6	rs2395670	37082809	0.011	A	G	1.3437002	1.1122	0.2280719
7	rs11764012	8207615	A	G	1348	0.6388	-4.427	9.56E-06	7	rs2058519	8214574	0.478	G	A	1.2305445	-0.0447	0.4622293
8	rs1870396	9261456	G	C	1333	1.741	5.065	4.08E-07	8	rs6601306	9264711	0.224	T	C	0.8644291	-1.1714	0.7694562
8	rs2256087	11496193	C	A	1380	0.5757	-4.552	5.32E-06	8	rs2256087	11496193	1	C	A	1.5806599	0.9401	0.116553
12	rs491825	113048587	T	C	1344	1.522	4.487	7.21E-06	12	rs17824050	113036817	0.553	A	G	0.8728475	0.4514	0.6146593
12	rs471231	118187337	A	G	1379	0.5948	-4.77	1.84E-06	12	rs471231	118187337	1	A	G	1.2764438	1.6962	0.3977656
17	rs75997523	6730523	A	G	1350	2.895	4.756	1.97E-06	17	rs12601392	6699944	0.537	G	A	1.2769892	0.4180	0.6042291
17	rs11656431	32392629	A	G	1327	5.12	4.576	4.74E-06	17	rs11658185	32394763	0.158	G	A	0.8852353	0.1711	0.7424022
18	rs9952569	24067227	C	T	1374	1.999	5.502	3.76E-08	18	rs9952569	24067227	1	C	T	0.5970534	-1.0367	0.2872871
20	rs6056644	9467164	C	A	1380	1.816	4.722	2.33E-06	20	rs6056647	9476953	0.976	A	G	1.4052022	0.8625	0.3847928
20	rs6044984	17718017	A	G	1374	0.5503	-4.645	3.40E-06	20	rs13042529	17698510	0.493	A	G	0.8046117	-1.4447	0.5881554
20	rs117452506	50185618	A	G	1336	2.385	4.448	8.68E-06	20	rs8121883	50197080	0.106	G	A	1.4445344	0.8726	0.2055499

The gene set enrichment analysis showed that the four protein-coding genes prioritized by FUMA (*AQP4*, *AQP4-AS*, *KCTD1* and *U3*) were statistically significantly enriched (p<0.000314; Bonferroni corrected for 3 * 53 tests) in overexpressed genes in hippocampal and caudate tissue from GTEx v6 (S5 Fig).

We tested the 17 top SNPs within each suggestive loci in the PING cohort for an association with IHI. In PING, there were either genotyped or SNPs in near perfect LD (r2>0.9) for seven loci, intermediate (0.25 < r2 < 0.9) proxies for five loci and weak proxies for the remaining five loci (r2 < 0.25). None of the selected candidate SNPs showed a nominal significant association (uncorrected p < 0.05) with IHI in this cohort. The top SNP from the discovery cohort, rs9952569, was not significant in the validation sample and showed an effect in the opposite direction (OR = 0.597; P = 0.29; Table 2).

### Heritability analysis and genetic correlation

Heritability of IHI was estimated from the GWAS summary statistics using LD score regression. The *h*^*2*^ estimate was 0.54 (0.30) and statistically significant using a one-sided test (Z = 1.8; P = 0.036). We next sought to identify genomics regions or cell type marker genes that show enriched heritability. However, none of the tested genomic regions or gene sets showed statistically significant enrichment after FDR correction (S6 Fig).

Motivated by reported increased prevalence of IHI in persons with epilepsy we computed the genetic correlation (rg) between IHI and epilepsy susceptibility. The estimate of rg was -0.0854 (0.2612) which did not reach statistically significance (Z = -0.3269; P = 0.7437).

LDhub was used to compute rg between IHI and 832 GWAS summary statistics; the computation was successful for 749 GWAS (S1 Table). None of the traits survived the FDR corrected p-value threshold (P_FDR_<0.05). A total of 20 traits reached nominal significance (p<0.05, Table 3). The top three positively correlated traits were: intelligence [58], College or University degree based on a UK BioBank (UKBB) GWAS and Years of Schooling [59]. Among the 20 nominal significant genetic correlations was also Fluid Intelligence Score (UKBB).

**Table 3:** Genetic correlations between IHI and other GWASs with nominal significance (p<0.05).

Trait	PMID	Category	rg	se	z	p
Intelligence	28530673	cognitive	0.3479	0.139	2.5037	0.0123
Qualifications: College or University degree	0	ukbb	0.3472	0.1392	2.494	0.0126
Years of schooling 2016	27225129	education	0.3227	0.13	2.4828	0.013
HDL cholesterol	20686565	lipids	0.3992	0.1742	2.2923	0.0219
Ulcerative colitis	26192919	autoimmune	0.4354	0.1978	2.2015	0.0277
Started insulin within one year diagnosis of diabetes	0	ukbb	0.7574	0.3488	2.1716	0.0299
Mothers age at death	0	ukbb	0.6082	0.2801	2.1717	0.0299
Medication for cholesterol_ blood pressure or diabetes: Insulin	0	ukbb	1.2001	0.5571	2.154	0.0312
Creatinine	27005778	metabolites	0.7254	0.3513	2.0652	0.0389
Pain type(s) experienced in last month: Back pain	0	ukbb	-0.3682	0.1826	-2.0165	0.0438
Illnesses of father: Chronic bronchitis/emphysema	0	ukbb	-0.4744	0.2357	-2.0132	0.0441
Triglycerides in small VLDL	27005778	metabolites	0.643	0.3196	2.0123	0.0442
Age at Menopause	26414677	reproductive	0.4687	0.2331	2.0103	0.0444
Fluid intelligence score	0	ukbb	0.2584	0.1286	2.0094	0.0445
Smoking/smokers in household	0	ukbb	-0.5759	0.2881	-1.9987	0.0456
Tinnitus: Yes_ now some of the time	0	ukbb	-1.3776	0.6904	-1.9954	0.046
Concentration of small VLDL particles	27005778	metabolites	0.6411	0.3221	1.9904	0.0465
Time spent using computer	0	ukbb	0.2483	0.1249	1.9883	0.0468
Pack years adult smoking as proportion of life span exposed to smoking PREVIEW ONLY	0	ukbb	-0.3127	0.1587	-1.9702	0.0488
Serum total triglycerides	27005778	metabolites	0.6408	0.3258	1.9671	0.0492

### Gene expression in the developing human brain

We extracted the gene expression levels in the developing brain for *AQP4* and *KCTD1*. The summary of the data up to post-conception week (pcw) 100 are depicted in S7 Fig. Expression of *KCTD1* remains rather stable across the entire time frame, while the expression of *AQP4* starts very low and increases with the progression of brain maturation and reaches its peak around the time of term birth (pcw 37–40). Gene expression was significantly different before and after pcw 25 for both *KCTD1* (p = 3.115e-04) and *AQP4* (p = 2.424e-07) when limited to data acquired before pcw 40.

## Discussion

Incidence rate of IHI was consistently around 25% in both, the discovery and the validation cohort. This was comparable to previous reports of 18–19% [60,61], especially considering that the IHI score at the 4.0 cutoff includes not only strong IHI (as in the cited studies), but also lighter IHI [15], therefore increasing the IHI rate. In both cohorts there was a higher incidence rate of IHI in the left hippocampus, which agrees well with the observations that the right hippocampus matures faster and thereby inverts correctly. The GWAS highlighted one genome-wide significant locus on chromosome 18, which is linked through chromatin interaction maps (S4 Fig, left) to six genes, two of which show substantial expression in brain tissue: *KCTD1* and *AQP4*. Of note, the locus showed consistently strong association with continuous scales of the IHI phenotype. Furthermore, a genome-wide screen of those continuous scales revealed two genome-wide significant loci (S3 Fig), one of which exceeded the suggestive threshold in the original GWAS (Table 2; rs35806781, OR = 4.889, Z = 4.798, P = 1.603e-06). The other SNP (rs186025034) had a low minor allele frequency (about 1%) and missed the suggestive threshold in the original GWAS (OR = 5.159, Z = 4.34, P = 1.423e-05). Overall, we observed consistency across IHI definitions and their genetic associations.

The Potassium Channel Tetramerization Domain Containing 1 (*KCTD1*) gene negatively regulates the AP-2 family of transcription factors and the Wnt signalling pathway, which controls normal embryonic development, cellular proliferation and growth [62]. Interestingly, mutations in *KCTD1* have been linked to Scalp-Ear-Nipple syndrome [63], which is a rare, autosomal-dominant disorder characterized by cutis aplasia of the scalp as well as minor anomalies of the external ears, digits, nails, and malformations of the breast. Clearly, *KCTD1* has the ability to influence developmental processes. Thus, it is conceivable that more benign variation in *KCTD1* may play a role in the generation of IHI. Furthermore, *KCTD1* is a potassium channel gene and various members of the potassium channel gene family have been linked as causes of epilepsy [64–66]. In a recent GWAS for epilepsy susceptibility SNPs in the *KCTD1* gene reached p-values as low as 0.0003758 (S8 Fig) [56].

Aquaporin-4 (*AQP4*) is a bidirectional water channel that is found on astrocytes throughout the central nervous system (S4 Fig). However, while *AQP4* expression in brain tissue is in general high in children and adults, its expression is quite low before post-conception week 20 (S7 Fig). MRI studies of IHI during brain development [20,23] show lack of hippocampal inversion during the early phases of development <25 post-conception weeks, which coincides with the time point of increased *AQP4* expression. Furthermore, *AQP4* has been linked through various lines of evidence to epilepsy, e.g., the lack of aquaporin-4 water channels increased seizure threshold and seizure duration in mice [67,68] and *AQP4* expression among chronic temporal lobe epilepsy patients is increased almost twofold in the hippocampus of the affected hemisphere compared to the contralateral hemisphere [69]. Taken together, astroglial *AQP4* may modulate neuronal excitability by regulating the extraneuronal and extrasynaptic environments and thereby affect the epileptogenesis. This may explain the observed increased rates of IHI in persons with epilepsy. Interestingly, the four protein-coding genes prioritized by FUMA were also enriched in genes overexpressed in hippocampal and caudate tissue (S5 Fig). However, enrichment results tend to be unstable when only a small gene set is tested for enrichment, thus, this result should be considered with caution regarding its interpretation.

We attempted to validate the genome-wide significant locus in a second independent cohort of adolescents. However, neither the genome-wide significant locus, nor any of the suggestive loci reached nominal significance in the validation cohort. One major contributor to this lack of replication was the limited sample size of the validation cohort. Despite the equally large set of participants in both studies, age and ethnicity restrictions severely limited the available sample size for the validation cohort (N = 161) and drastically lowered the statistical power to detect differences (power of 35% for just the top variant). Larger validation cohorts are needed to confirm the association of the identified locus with IHI: e.g., to validate the top variant with 80% at least N = 500 subjects are required. Although there are growing imaging and genetic datasets, e.g., the UKBB that aims at 100,000 participants with genetics and brain imaging data, few studies focus on healthy younger subjects (children, adolescents or young adults), which is beneficial for the validation in order to exclude confounding by disease processes or age-related atrophy. One such option is the Philadelphia Neurodevelopmental Cohort (PNC) [70]. However, it is important to keep in mind that the evaluation of IHI is not restricted to adolescents. IHI can be observed in children and adults too, without extra difficulties. The study here focus on adolescents, since the discovery cohort is the dataset used for the reference study [15]. Still, in older patients, even though IHI still exist, their detection may be more difficult and less reliable due to the confounding effect of hippocampal atrophy to ageing, hippocampal sclerosis or neurodegeneration. Also scoring bigger databases (such as UKBB) will be feasible only after automatic methods for IHI scoring have been developed.

We estimated heritability of IHI based on the state-of-the-art LD score regression method that operates on the GWAS summary statistics. The inferred heritability was substantial with *h*^*2*^ = 0.54; the estimate was subject to high uncertainty as reflected by the high standard deviation of 0.3, which is likely a direct reflection of the low sample size of the discovery cohort. Analysis on twin data, such as the healthy young adult twins participating in the Queensland Twin IMaging (QTIM) study [71], can be used to confirm this preliminary heritability estimate, but would require a significant effort in manually scoring IHI in these large cohorts. In addition, the magnitude of *h*^*2*^ is comparable to recently published estimates on the heritability of hippocampal volume and shape from more than 3600 subjects [25]; *h*^*2*^ ranges from 0.08 to 0.337 depending on hemisphere and structural measure, with heritability of volume being generally the lowest. Given the impact of IHI on hippocampal shape and appearance together with the high prevalence of IHI in nearly 25% of healthy subjects, it is likely that the observed heritability of hippocampal shape reported by [25] was in part due to IHI.

We used the generated genome-wide summary statistics for two additional explorations. First, we sought to investigate if heritability was enriched in any particular region of the genome, characterized by its function or by marker genes for specific cell types. None of the investigated categories achieved statistical significance after FDR correction. Second, we computed genetic correlations with other traits. We hypothesized that there may be genetic link with epilepsy, however the resulting correlation was non-significant and negative, i.e., people with IHI were less likely to be affected by epilepsy, thereby contradicting earlier reports. The exploratory analysis with 832 additional traits highlighted a positive genetic correlation between IHI and intelligence and education attainment. There are various reports highlighting the contribution of the hippocampus and its subregions to various mental aspects that collectively are referred to as intelligence, e.g., spatial processing [72] and working memory [73]. Moreover, one recent study linked hippocampal shape to cognitive performance [74]. In particular, in males the radial distance of the hippocampus correlated with better test scores (e.g., general factor of intelligence, abstract-fluid intelligence, and the rotation of solid figures). In females, the effect was reversed. Therefore, a genetic correlation of IHI with intelligence in the broad sense is conceivable.

In conclusion, we presented the first genome-wide association study of IHI, where we identified a genome-wide significant locus. Additional exploration of the resulting summary statistics revealed a high heritability and suggested positive genetic correlation of IHI with traits linked to intelligence and education attainment.

## Ethics committee

London: Psychiatry, Nursing and Midwifery (PNM) Research Ethics Subcommittee (RESC), Waterloo Campus, King's College London. Nottingham: University of Nottingham Medical School Ethics Committee. Mannheim: Medizinische Fakultaet Mannheim, Ruprecht Karl Universitaet Heidelberg and Ethik-Kommission II an der Fakultaet fuer Kliniksche Medizin Mannheim. Dresden: Ethikkommission der Medizinischen Fakultaet Carl Gustav Carus, TU Dresden Medizinische Fakultaet. Hamburg: Ethics board, Hamburg Chamber of Phsyicians. Paris: CPP IDF VII (Comité de protection des personnes Ile de France), ID RCB: 2007-A00778-45 September 24th 2007. Dublin: TCD School of Psychology REC. Berlin: ethics committee of the Faculty of Psychology. And Mannheim's ethics committee approved the whole study. All informed consents have been obtained from a parent and/or legal guardian. Written informed assent and consent were obtained, respectively, from all adolescents and their parents after complete description of the study. For the PING dataset, written parental informed consent was obtained for all PING subjects below the age of 18, and child assent was also obtained for all participants between the ages of 7 and 17. Written informed consent was obtained directly from all participants aged 18 years or older.

## Supporting information

S1 Fig Ancestry matching of the IMAGEN cohort. The panels show pairwise scatter plots for the first five principal components of the genetic relationship matrix. The HapMap3 subjects are represented as filled circles and color coded by population (see legend in the top left panel). IMAGEN participants are represented by black open circles; red open circles indicate subjects that were excluded based on distance to the CEU+TSI European ancestry.
(TIF)

S2 Fig Quantile-quantile plot. QQ plot for the genome-wide association study. The xaxis shows the expected -log10(p-value) while the y-axis shows the study -log10(p-value). There was no evidence of p-value inflation λ = 1.017.
(TIF)

S3 Fig Manhattan plots for continuous IHI scores. The y-axis depicts the -log10(p-value) of the association between SNP and presence of IHI assuming an additive model in the discovery cohort. The SNPs tested in the study are ordered along their chromosomal position on the x-axis. The red horizontal line donates genome wide significance at the Bonferroni threshold (P = 5e-8), while the blue horizontal line marks the threshold for suggestive association (P = 1e-5). Upper plot: Result for the sum of the five criteria and then maxed over left and right hippocampus. Two loci exceed the genome wide significant threshold: on chromosome 6 rs35806781 (beta = 1.478, Z = 5.766, P = 1.006e-08) and on chromosome 9 rs186025034 (beta = 1.867, Z = 6.202, P = 7.408e-10). Lower plot: Result for the global criterion presenting 3 classes: 0 = non IHI, 2 = IHI, 1 = partial IHI, and taking the max over left and right hippocampus. One locus exceeds genome-wide significance on chromosome 9: rs186025034 (beta = 0.8251, Z = 5.575, P = 2.98e-08).
(TIF)

S4 Fig FUMA results. Left: chromatin interaction plot, mapping the genome-wide significant locus to six genes on chromosome 18. Right: expression heatmap (average log2(RPKM) in 53 GTEx tissues) for the four mapped protein-coding genes.
(TIF)

S5 Fig Enrichment analysis of prioritized protein-coding genes in GTEx. The four prioritized genes are tested for enrichment in tissue-specific gene lists for 53 tissue from the GTEx dataset. Each list of differentially expressed genes is split into overexpressed and underexpressed genes. P-value threshold for significance was the Bonferroni corrected threshold for 3 * 53 tests (p<0.000314). Tissues showing significant enrichment are indicated by red bars.
(TIF)

S6 Fig Heritability partitioning analysis: Panels 1–5, among cell/tissue-specific differentially expressed genes; panel 6, among genomic functional annotation categories. (TIF)

S7 Fig Gene expression in the developing human brain for AQP4 and KCTD1. The x-axis shows the subjects’ age in weeks post conception (pcw). The y-axis depicts the mean of the log2 RPKM (reads per kilobase per million) provided by BrainSpan. AQP4 and KCTD1 are indicated by different colors.
(TIF)

S8 Fig Local association plot for KCTD1 in ILAE epilepsy GWAS. (TIF)

S1 Table Genetic correlation between IHI and 832 GWASs obtained from LD hub. (CSV)

S1 File Imagen Consortium author list. (DOCX)

## References

[1] Burgess N, Maguire EA, O’Keefe J. The Human Hippocampus and Spatial and Episodic Memory. Neuron. 2002 8;35[4]:625–41. 10.1016/s0896-6273(02)00830-9 12194864

[2] Jankord R, Herman JP. Limbic Regulation of Hypothalamo-Pituitary-Adrenocortical Function during Acute and Chronic Stress. Annals of the New York Academy of Sciences. 2008 12;1148[1]:64–73. 19120092 10.1196/annals.1410.012 PMC2637449

[3] Barnes J, Bartlett JW, van de Pol LA, Loy CT, Scahill RI, Frost C, et al A meta-analysis of hippocampal atrophy rates in Alzheimer’s disease. Neurobiology of Aging. 2009 11;30[11]:1711–23. 10.1016/j.neurobiolaging.2008.01.010 18346820 PMC2773132

[4] Colle R, Cury C, Chupin M, Deflesselle E, Hardy P, Nasser G, et al Hippocampal volume predicts antidepressant efficacy in depressed patients without incomplete hippocampal inversion. NeuroImage: Clinical. 2016 2 1;12:949–55. 27995060 10.1016/j.nicl.2016.04.009 PMC5153557

[5] Sämann PG, Höhn D, Chechko N, Kloiber S, Lucae S, Ising M, et al Prediction of antidepressant treatment response from gray matter volume across diagnostic categories. European Neuropsychopharmacology. 2013 11;23[11]:1503–15. 10.1016/j.euroneuro.2013.07.004 23920122

[6] Colle R, Chupin M, Cury C, Vandendrie C, Gressier F, Hardy P, et al Depressed suicide attempters have smaller hippocampus than depressed patients without suicide attempts. Journal of Psychiatric Research. 2015 2;61:13–8. 10.1016/j.jpsychires.2014.12.010 25555305

[7] Travis SG, Coupland NJ, Hegadoren K, Silverstone PH, Huang Y, Carter R, et al Effects of cortisol on hippocampal subfields volumes and memory performance in healthy control subjects and patients with major depressive disorder. Journal of Affective Disorders. 2016 9;201:34–41. 10.1016/j.jad.2016.04.049 27162154

[8] van Erp TGM, Hibar DP, Rasmussen JM, Glahn DC, Pearlson GD, Andreassen OA, et al Subcortical brain volume abnormalities in 2028 individuals with schizophrenia and 2540 healthy controls via the ENIGMA consortium. Mol Psychiatry. 2016 4;21[4]:547–53. 10.1038/mp.2015.63 26033243 PMC4668237

[9] Helmstaedter C, Elger CE. Chronic temporal lobe epilepsy: a neurodevelopmental or progressively dementing disease? Brain. 2009 10 1;132[10]:2822–30. 19635728 10.1093/brain/awp182

[10] Qiu A, Rifkin-Graboi A, Chen H, Chong Y-S, Kwek K, Gluckman PD, et al Maternal anxiety and infants’ hippocampal development: timing matters. Transl Psychiatry. 2013 9;3[9]:e306–e306. 24064710 10.1038/tp.2013.79 PMC3784768

[11] Bronen RA, Cheung G. MRI of the normal hippocampus. Magnetic Resonance Imaging. 1991;9[4]:497–500. 10.1016/0730-725x(91)90035-k 1779720

[12] Lehéricy S, Dormont D, Sémah F, Clémenceau S, Granat O, Marsault C, et al Developmental abnormalities of the medial temporal lobe in patients with temporal lobe epilepsy. AJNR American journal of neuroradiology. 1995;16[4]:617–26. 7611013 PMC8332297

[13] Baulac M, De Grissac N, Hasboun D, Oppenheim C, Adam C, Arzimanoglou A, et al Hippocampal developmental changes in patients with partial epilepsy: magnetic resonance imaging and clinical aspects. Annals of neurology. 1998;44:223–33. 10.1002/ana.410440213 9708545

[14] Bernasconi N, Kinay D, Andermann F, Antel S, Bernasconi A. Analysis of shape and positioning of the hippocampal formation: an MRI study in patients with partial epilepsy and healthy controls. Brain: a journal of neurology. 2005;128:2442–2452. 16014649 10.1093/brain/awh599

[15] Cury C, Toro R, Cohen F, Fischer C, Mhaya A, Samper-González J, et al Incomplete Hippocampal Inversion: A Comprehensive MRI Study of Over 2000 Subjects. Frontiers in neuroanatomy. 2015;9:160 10.3389/fnana.2015.00160 26733822 PMC4686650

[16] Cury C. Statistical shape analysis of the anatomical variability of the human hippocampus in large populations Sorbonne Université; 2015.

[17] Colenutt J, McCann B, Knight MJ, Coulthard E, Kauppinen RA. Incomplete Hippocampal Inversion and Its Relationship to Hippocampal Subfield Volumes and Aging: Incomplete Hippocampal Inversion and Aging. Journal of Neuroimaging. 2018 7;28[4]:422–8. 29575376 10.1111/jon.12509

[18] Kim H, Chupin M, Colliot O, Bernhardt BC, Bernasconi N, Bernasconi A. Automatic hippocampal segmentation in temporal lobe epilepsy: Impact of developmental abnormalities. NeuroImage. 2012 2 15;59[4]:3178–86. 10.1016/j.neuroimage.2011.11.040 22155377

[19] Okada Y, Kato T, Iwai K, Iwasaki N, Ohto T, Matsui A. Evaluation of hippocampal infolding using magnetic resonance imaging. NeuroReport. 2003;14[10]:1405–9. 10.1097/01.wnr.0000078381.40088.d0 12876483

[20] Bajic D, Ewald U, Raininko R. Hippocampal development at gestation weeks 23 to 36. An ultrasound study on preterm neonates. Neuroradiology. 2010;52[6]:489–94. 10.1007/s00234-010-0673-x 20352419

[21] Baker LL, Barkovich AJ. The large temporal horn: MR analysis in developmental brain anomalies versus hydrocephalus. American Journal of Neuroradiology. 1992;13[1]:115–22. 1595428 PMC8331781

[22] Righini A, Zirpoli S, Parazzini C, Bianchini E, Scifo P, Sala C, et al Hippocampal infolding angle changes during brain development assessed by prenatal MR imaging. American Journal of Neuroradiology. 2006;27[10]:2093–7. 17110674 PMC7977211

[23] Bajic D, Canto Moreira N, Wikström J, Raininko R. Asymmetric development of the hippocampal region is common: A fetal MR imaging study. American Journal of Neuroradiology. 2012;33[3]:513–8. 22116115 10.3174/ajnr.A2814 PMC7966435

[24] Andrade D, Krings T, Chow EWC, Kiehl TR, Bassett AS. Hippocampal malrotation is associated with chromosome 22q11.2 microdeletion. Canadian Journal of Neurological Sciences. 2013;40[5]:652–6. 10.1017/s0317167100014876 23968937 PMC4459860

[25] Roshchupkin G V., Gutman BA, Vernooij MW, Jahanshad N, Martin NG, Hofman A, et al Heritability of the shape of subcortical brain structures in the general population. Nature Communications. 2016;7:13738 10.1038/ncomms13738 27976715 PMC5172387

[26] Schumann G, Loth E, Banaschewski T, Barbot A, Barker G, Büchel C, et al The IMAGEN study: reinforcement-related behaviour in normal brain function and psychopathology. Molecular psychiatry. 2010;15[12]:1128–39. 10.1038/mp.2010.4 21102431

[27] Jernigan TL, Brown TT, Hagler DJ, Akshoomoff N, Bartsch H, Newman E, et al The Pediatric Imaging, Neurocognition, and Genetics (PING) Data Repository. NeuroImage. 2016;124[Pt B]:1149–54. 10.1016/j.neuroimage.2015.04.057 25937488 PMC4628902

[28] Jenkinson M, Smith S. A global optimisation method for robust affine registration of brain images. Medical Image Analysis. 2001;5[2]:143–56. 10.1016/s1361-8415(01)00036-6 11516708

[29] Jenkinson M, Bannister P, Brady M, Smith S. Improved optimization for the robust and accurate linear registration and motion correction of brain images. NeuroImage. 2002;17[2]:825–41. 10.1016/s1053-8119(02)91132-8 12377157

[30] The International HapMap 3 Consortium, Altshuler DM, Gibbs RA, Peltonen L, Dermitzakis E, Schaffner SF, et al Integrating common and rare genetic variation in diverse human populations. Nature. 2010;467:52–58. 10.1038/nature09298 20811451 PMC3173859

[31] Yang J, Lee SH, Goddard ME, Visscher PM. GCTA: a tool for genome-wide complex trait analysis. American journal of human genetics. 2011;88[1]:76–82. 10.1016/j.ajhg.2010.11.011 21167468 PMC3014363

[32] McCarthy S, Das S, Kretzschmar W, Delaneau O, Wood AR, Teumer A, et al A reference panel of 64,976 haplotypes for genotype imputation. Nature Genetics. 2016;48:1279–1283. 10.1038/ng.3643 27548312 PMC5388176

[33] Loh PR, Danecek P, Palamara PF, Fuchsberger C, Reshef YA, Finucane HK, et al Reference-based phasing using the Haplotype Reference Consortium panel. Nature Genetics. 2016;48[11]:1443–8. 10.1038/ng.3679 27694958 PMC5096458

[34] Durbin R. Efficient haplotype matching and storage using the positional Burrows-Wheeler transform (PBWT). Bioinformatics. 2014;30[9]:1266–72. 10.1093/bioinformatics/btu014 24413527 PMC3998136

[35] Alexander DH, Novembre J, Lange K. Fast model-based estimation of ancestry in unrelated individuals. Genome Research. 2009;19[9]:1655–1664. 10.1101/gr.094052.109 19648217 PMC2752134

[36] Chang CC, Chow CC, Tellier LCAM, Vattikuti S, Purcell SM, Lee JJ. Second-generation PLINK: Rising to the challenge of larger and richer datasets. GigaScience. 2015;4[1]. 10.1186/s13742-015-0047-8 PMC4342193 25722852

[37] Pruim RJ, Welch RP, Sanna S, Teslovich TM, Chines PS, Gliedt TP, et al LocusZoom: regional visualization of genome-wide association scan results. Bioinformatics. 2010;26[18]:2336–7. 10.1093/bioinformatics/btq419 20634204 PMC2935401

[38] Machiela MJ, Chanock SJ. LDlink: A web-based application for exploring population-specific haplotype structure and linking correlated alleles of possible functional variants. Bioinformatics. 2015;31[21]:3555–7. 10.1093/bioinformatics/btv402 26139635 PMC4626747

[39] Watanabe K, Taskesen E, van Bochoven A, Posthuma D. Functional mapping and annotation of genetic associations with FUMA. Nature communications. 2017;8[1]:1826 10.1038/s41467-017-01261-5 29184056 PMC5705698

[40] Consortium GTEx. Human genomics. The Genotype-Tissue Expression (GTEx) pilot analysis: multitissue gene regulation in humans. Science (New York, NY). 2015;348[6235]:648–60. 10.1126/science.1262110 PMC4547484 25954001

[41] Westra HJ, Peters MJ, Esko T, Yaghootkar H, Schurmann C, Kettunen J, et al Systematic identification of trans eQTLs as putative drivers of known disease associations. Nature Genetics. 2013;45:1238–1243. 10.1038/ng.2756 24013639 PMC3991562

[42] Zhernakova D V., Deelen P, Vermaat M, Van Iterson M, Van Galen M, Arindrarto W, et al Identification of context-dependent expression quantitative trait loci in whole blood. Nature Genetics. 2017;49:139–145. 10.1038/ng.3737 27918533

[43] Ramasamy A, Trabzuni D, Guelfi S, Varghese V, Smith C, Walker R, et al Genetic variability in the regulation of gene expression in ten regions of the human brain. Nature neuroscience. 2014;17[10]:1418–28. 10.1038/nn.3801 25174004 PMC4208299

[44] Schmitt AD, Hu M, Jung I, Xu Z, Qiu Y, Tan CL, et al A Compendium of Chromatin Contact Maps Reveals Spatially Active Regions in the Human Genome. Cell Reports. 2016;17:2042–2059. 10.1016/j.celrep.2016.10.061 27851967 PMC5478386

[45] Roadmap Epigenomics Consortium, Kundaje A, Meuleman W, Ernst J, Bilenky M, Yen A, et al Integrative analysis of 111 reference human epigenomes. Nature. 2015;518:317–330. 10.1038/nature14248 25693563 PMC4530010

[46] Liberzon A, Subramanian A, Pinchback R, Thorvaldsdóttir H, Tamayo P, Mesirov JP. Molecular signatures database (MSigDB) 3.0. Bioinformatics. 2011;27:1739–1740. 10.1093/bioinformatics/btr260 21546393 PMC3106198

[47] Kutmon M, Riutta A, Nunes N, Hanspers K, Willighagen EL, Bohler A, et al WikiPathways: Capturing the full diversity of pathway knowledge. Nucleic Acids Research. 2016;44:D488–D494. 10.1093/nar/gkv1024 26481357 PMC4702772

[48] Welter D, MacArthur J, Morales J, Burdett T, Hall P, Junkins H, et al The NHGRI GWAS Catalog, a curated resource of SNP-trait associations. Nucleic Acids Research. 2014;42:D1001–D1006. 10.1093/nar/gkt1229 24316577 PMC3965119

[49] Bulik-Sullivan B, Loh P, Finucane H, Ripke S. LD Score regression distinguishes confounding from polygenicity in genome-wide association studies. Nature. 2015; 10.1038/ng.3211 PMC4495769 25642630

[50] Finucane HK, Bulik-Sullivan B, Gusev A, Trynka G, Reshef Y, Loh P-R, et al Partitioning heritability by functional annotation using genome-wide association summary statistics. Nature genetics. 2015;47[11]:1228–35. 10.1038/ng.3404 26414678 PMC4626285

[51] Finucane HK, Reshef YA, Anttila V, Slowikowski K, Gusev A, Byrnes A, et al Heritability enrichment of specifically expressed genes identifies disease-relevant tissues and cell types. Nature genetics. 2018;50[4]:621–9. 10.1038/s41588-018-0081-4 29632380 PMC5896795

[52] Cahoy JD, Emery B, Kaushal A, Foo LC, Zamanian JL, Christopherson KS, et al A transcriptome database for astrocytes, neurons, and oligodendrocytes: a new resource for understanding brain development and function. The Journal of neuroscience: the official journal of the Society for Neuroscience. 2008;28[1]:264–78. 18171944 10.1523/JNEUROSCI.4178-07.2008 PMC6671143

[53] Pers TH, Karjalainen JM, Chan Y, Westra H-J, Wood AR, Yang J, et al Biological interpretation of genome-wide association studies using predicted gene functions. Nature communications. 2015;6:5890 10.1038/ncomms6890 25597830 PMC4420238

[54] Heng TSP, Painter MW, Consortium IGP. The Immunological Genome Project: networks of gene expression in immune cells. Nature immunology. 2008;9[10]:1091–4. 10.1038/ni1008-1091 18800157

[55] Bulik-Sullivan B, Finucane HK, Anttila V, Gusev A, Day FR, Loh P-R, et al An atlas of genetic correlations across human diseases and traits. Nature genetics. 2015;47[11]:1236–41. 10.1038/ng.3406 26414676 PMC4797329

[56] The International League Against Epilepsy Consortium on Complex Epilepsies. Genetic determinants of common epilepsies: a meta-analysis of genome-wide association studies. Lancet neurology. 2014;13[9]:893–903. 10.1016/S1474-4422(14)70171-1 25087078 PMC4189926

[57] Zheng J, Erzurumluoglu AM, Elsworth BL, Kemp JP, Howe L, Haycock PC, et al LD Hub: a centralized database and web interface to perform LD score regression that maximizes the potential of summary level GWAS data for SNP heritability and genetic correlation analysis. Bioinformatics. 2017;33[2]:272–9. 10.1093/bioinformatics/btw613 27663502 PMC5542030

[58] Sniekers S, Stringer S, Watanabe K, Jansen PR, Coleman JRI, Krapohl E, et al Genome-wide association meta-analysis of 78,308 individuals identifies new loci and genes influencing human intelligence. Nature Genetics. 2017;49[7]:1107–12. 10.1038/ng.3869 28530673 PMC5665562

[59] Okbay A, Beauchamp JP, Fontana MA, Lee JJ, Pers TH, Rietveld CA, et al Genome-wide association study identifies 74 loci associated with educational attainment. Nature. 2016;533[7604]:539–42. 10.1038/nature17671 27225129 PMC4883595

[60] Bajic D, Wang C, Kumlien E, Mattsson P, Lundberg S, Eeg-Olofsson O, et al Incomplete inversion of the hippocampus—A common developmental anomaly. European Radiology. 2008;18[1]:138–42. 10.1007/s00330-007-0735-6 17828540

[61] Bajic D, Kumlien E, Mattsson P, Lundberg S, Wang C, Raininko R. Incomplete hippocampal inversion—Is there a relation to epilepsy? European Radiology. 2009;19[10]:2544–50. 10.1007/s00330-009-1438-y 19440714

[62] Li X, Chen C, Wang F, Huang W, Liang Z, Xiao Y, et al KCTD1 Suppresses Canonical Wnt Signaling Pathway by Enhancing β-catenin Degradation. PLoS ONE. 2014;9[4]:e94343 10.1371/journal.pone.0094343 24736394 PMC3988066

[63] Marneros AG, Beck AE, Turner EH, McMillin MJ, Edwards MJ, Field M, et al Mutations in KCTD1 cause scalp-ear-nipple syndrome. American Journal of Human Genetics. 2013;92[4]:621–6. 10.1016/j.ajhg.2013.03.002 23541344 PMC3617379

[64] Singh NA, Charlier C, Stauffer D, DuPont BR, Leach RJ, Melis R, et al A novel potassium channel gene, KCNQ2, is mutated in an inherited epilepsy of newborns. Nature Genetics. 1998;18:25–29. 10.1038/ng0198-25 9425895

[65] Jentsch TJ. Neuronal KCNQ potassium channels: physiology and role in disease. Nature reviews Neuroscience. 2000;1:21–30. 10.1038/35036198 11252765

[66] Liu Z, Xiang Y, Sun G. The KCTD family of proteins: structure, function, disease relevance. Cell & bioscience. 2013;3[45]. 10.1186/2045-3701-3-45 PMC3882106 24268103

[67] Binder DK, Yao X, Verkman AS, Manley GT. Increased seizure duration in mice lacking aquaporin-4 water channels. Acta Neurochirurgica, Supplementum. 2006;96. 10.1007/3-211-30714-1_80 16671491

[68] Binder DK, Oshio K, Ma T, Verkman AS, Manley GT. Increased seizure threshold in mice lacking aquaporin-4 water channels. NeuroReport. 2004;15[2]:259–62. 10.1097/00001756-200402090-00009 15076748

[69] Das A, Wallace GC, Holmes C, McDowell ML, Smith JA, Marshall JD, et al Hippocampal tissue of patients with refractory temporal lobe epilepsy is associated with astrocyte activation, inflammation, and altered expression of channels and receptors. Neuroscience. 2012;220:237–46. 10.1016/j.neuroscience.2012.06.002 22698689 PMC3412889

[70] Satterthwaite TD, Elliott MA, Ruparel K, Loughead J, Prabhakaran K, Calkins ME, et al Neuroimaging of the Philadelphia neurodevelopmental cohort. NeuroImage. 2014;86:544–53. 10.1016/j.neuroimage.2013.07.064 23921101 PMC3947233

[71] de Zubicaray GI, Chiang M-C, McMahon KL, Shattuck DW, Toga AW, Martin NG, et al Meeting the Challenges of Neuroimaging Genetics. 2008;2[4]:258–63. 10.1007/s11682-008-9029-0 PMC2794202 20016769

[72] Maguire EA, Gadian DG, Johnsrude IS, Good CD, Ashburner J, Frackowiak RSJ, et al Navigation-related structural change in the hippocampi of taxi drivers. Proceedings of the National Academy of Sciences. 2000;97[8]:4398–403. 10.1073/pnas.070039597 PMC18253 10716738

[73] Axmacher N, Henseler MM, Jensen O, Weinreich I, Elger CE, Fell J. Cross-frequency coupling supports multi-item working memory in the human hippocampus. Proceedings of the National Academy of Sciences. 2010;107[7]:3228–33. 10.1073/pnas.0911531107 PMC2840289 20133762

[74] Colom R, Stein JL, Rajagopalan P, Martínez K, Hermel D, Wang Y, et al Hippocampal structure and human cognition: Key role of spatial processing and evidence supporting the efficiency hypothesis in females. Intelligence. 2013;41[2]:129–140. 10.1016/j.intell.2013.01.002 25632167 PMC4306583

