# Supplementary figures and images for: Genome wide association study of incomplete hippocampal inversion in adolescents
Source: PLoS One. 2020 Jan 28;15(1):e0227355. doi: 10.1371/journal.pone.0227355 (PMC6986744; doi:10.1371/journal.pone.0227355)

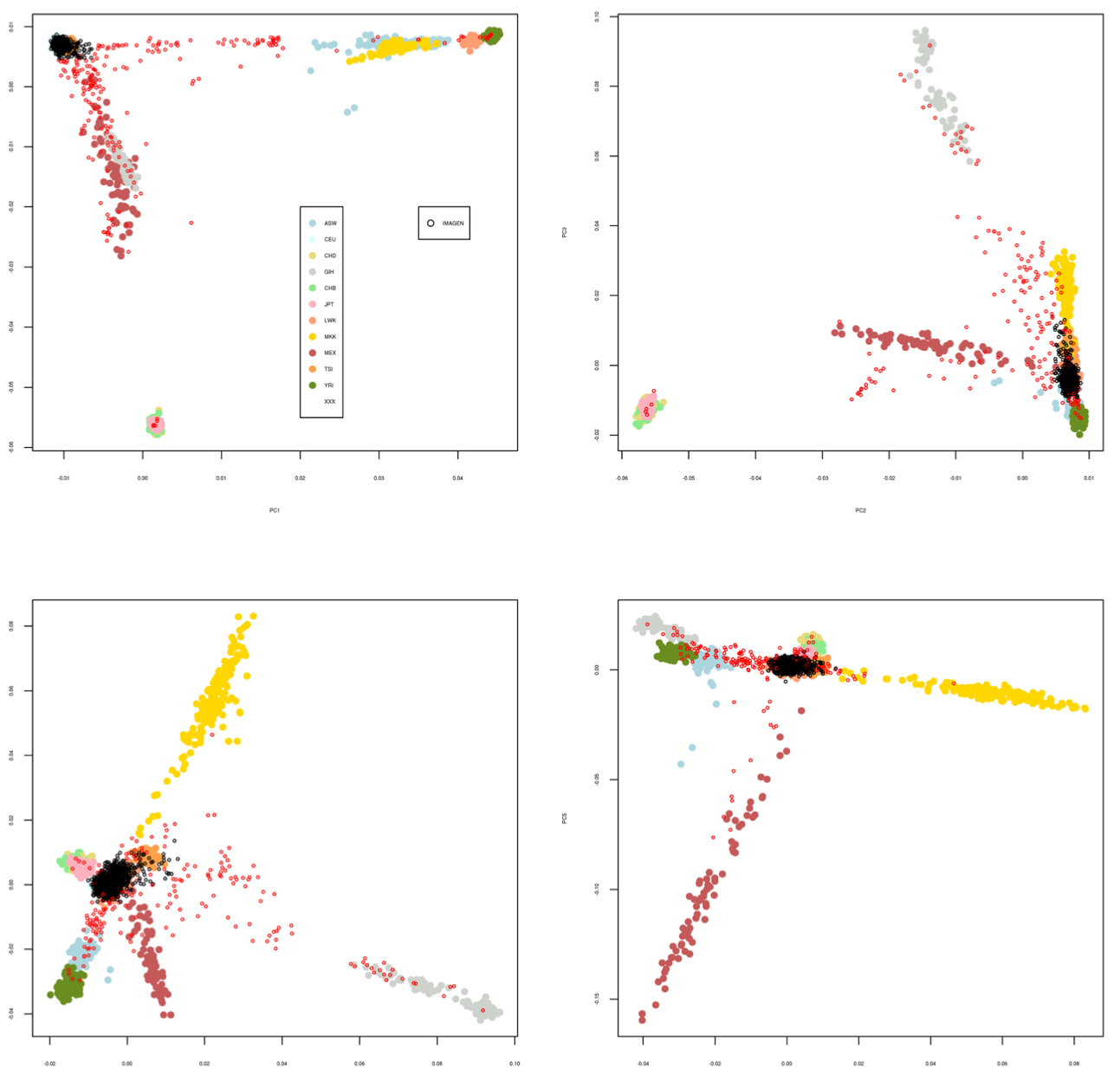

Supplement: S1 Fig — The panels show pairwise scatter plots for the first five principal components of the genetic relationship matrix. The HapMap3 subjects are represented as filled circles and color coded by population (see legend in the top left panel). IMAGEN participants are represented by black open circles; red open circles indicate subjects that were excluded based on distance to the CEU+TSI European ancestry. (TIF) [file pone.0227355.s001.tif]

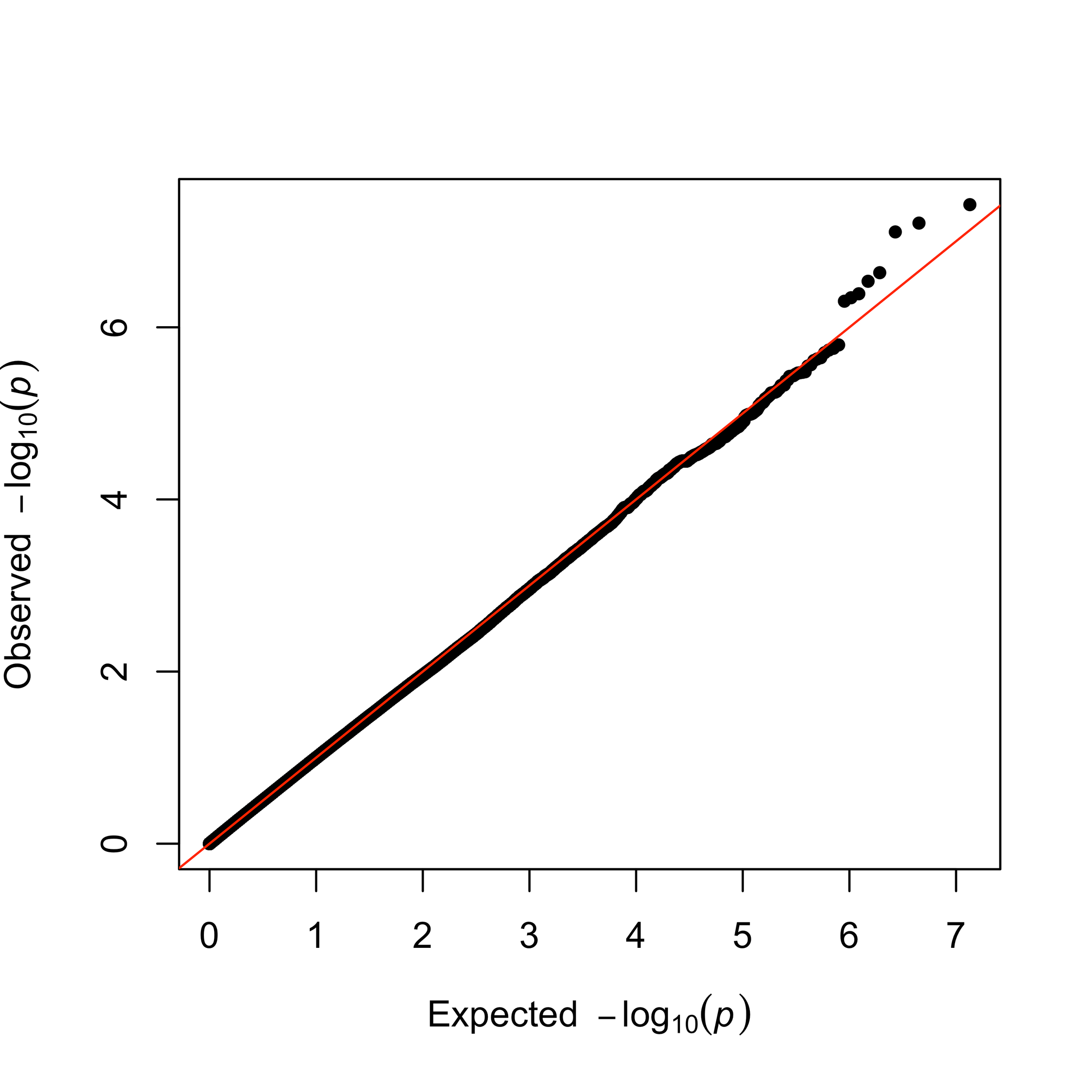

Supplement: S2 Fig — QQ plot for the genome-wide association study. The xaxis shows the expected -log10(p-value) while the y-axis shows the study -log10(p-value). There was no evidence of p-value inflation λ = 1.017. (TIF) [file pone.0227355.s002.tif]

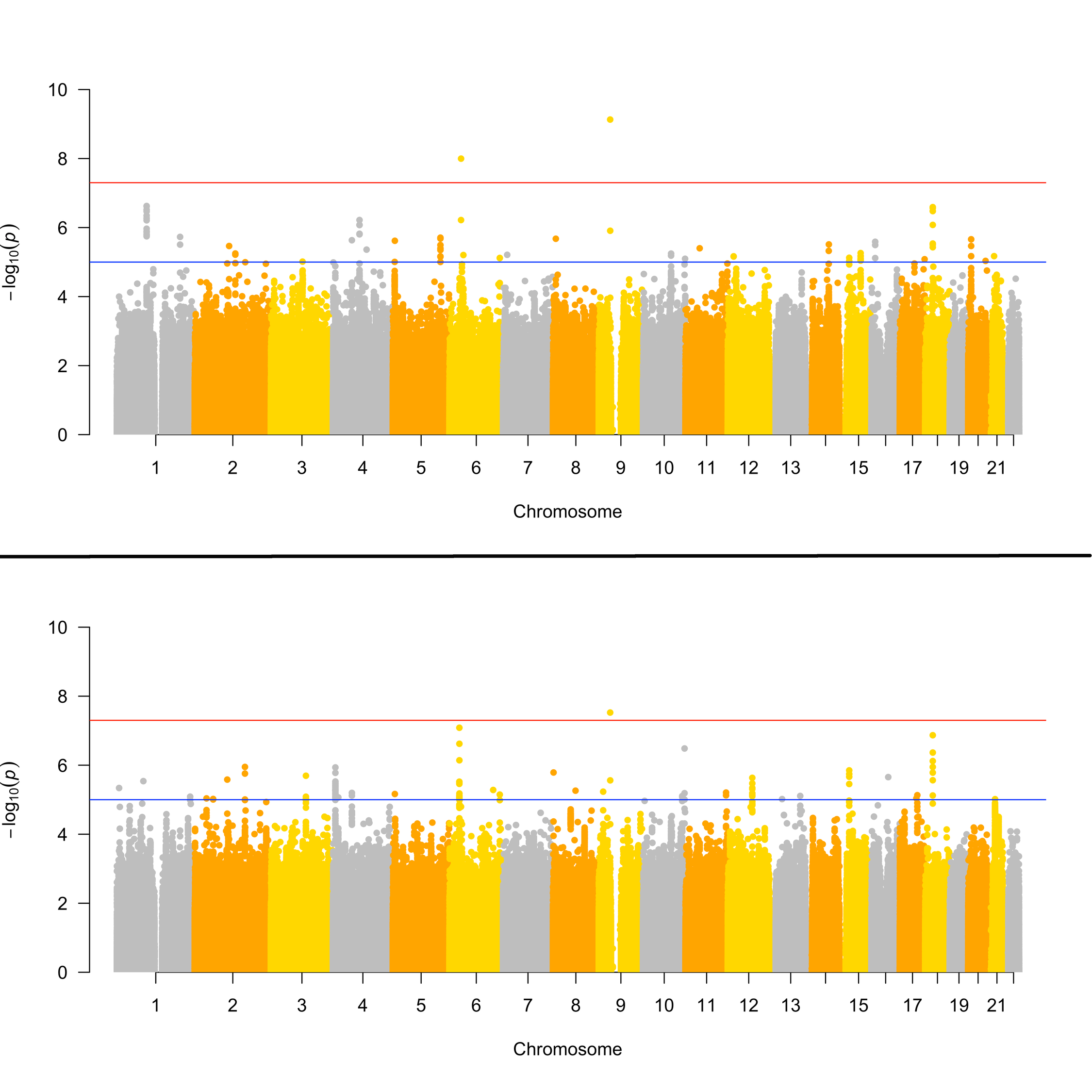

Supplement: S3 Fig — The y-axis depicts the -log10(p-value) of the association between SNP and presence of IHI assuming an additive model in the discovery cohort. The SNPs tested in the study are ordered along their chromosomal position on the x-axis. The red horizontal line donates genome wide significance at the Bonferroni threshold (P = 5e-8), while the blue horizontal line marks the threshold for suggestive association (P = 1e-5). Upper plot: Result for the sum of the five criteria and then maxed over left and right hippocampus. Two loci exceed the genome wide significant threshold: on chromosome 6 rs35806781 (beta = 1.478, Z = 5.766, P = 1.006e-08) and on chromosome 9 rs186025034 (beta = 1.867, Z = 6.202, P = 7.408e-10). Lower plot: Result for the global criterion presenting 3 classes: 0 = non IHI, 2 = IHI, 1 = partial IHI, and taking the max over left and right hippocampus. One locus exceeds genome-wide significance on chromosome 9: rs186025034 (beta = 0.8251, Z = 5.575, P = 2.98e-08). (TIF) [file pone.0227355.s003.tif]

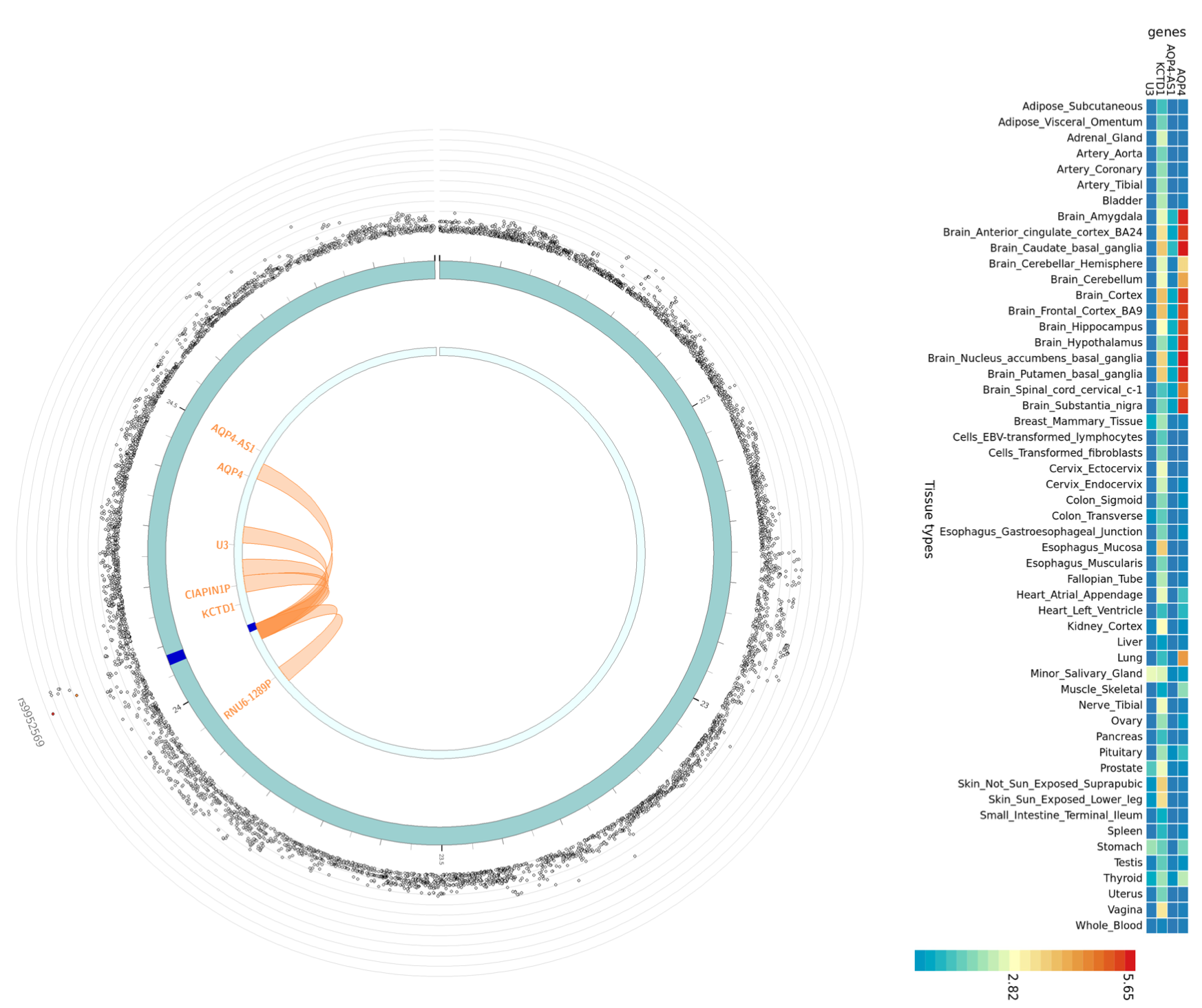

Supplement: S4 Fig — Left: chromatin interaction plot, mapping the genome-wide significant locus to six genes on chromosome 18. Right: expression heatmap (average log2(RPKM) in 53 GTEx tissues) for the four mapped protein-coding genes. (TIF) [file pone.0227355.s004.tif]

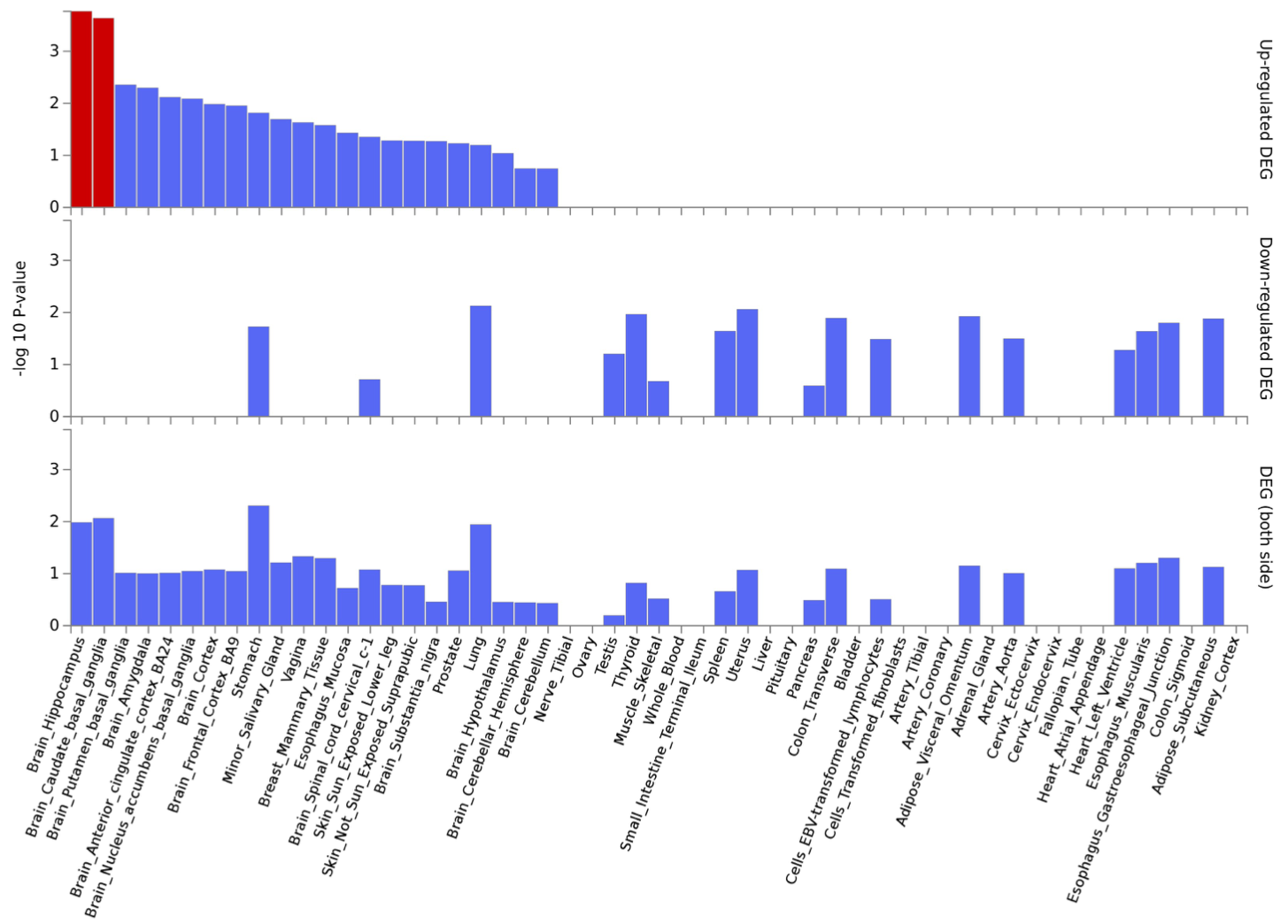

Supplement: S5 Fig — The four prioritized genes are tested for enrichment in tissue-specific gene lists for 53 tissue from the GTEx dataset. Each list of differentially expressed genes is split into overexpressed and underexpressed genes. P-value threshold for significance was the Bonferroni corrected threshold for 3 * 53 tests (p<0.000314). Tissues showing significant enrichment are indicated by red bars. (TIF) [file pone.0227355.s005.tif]

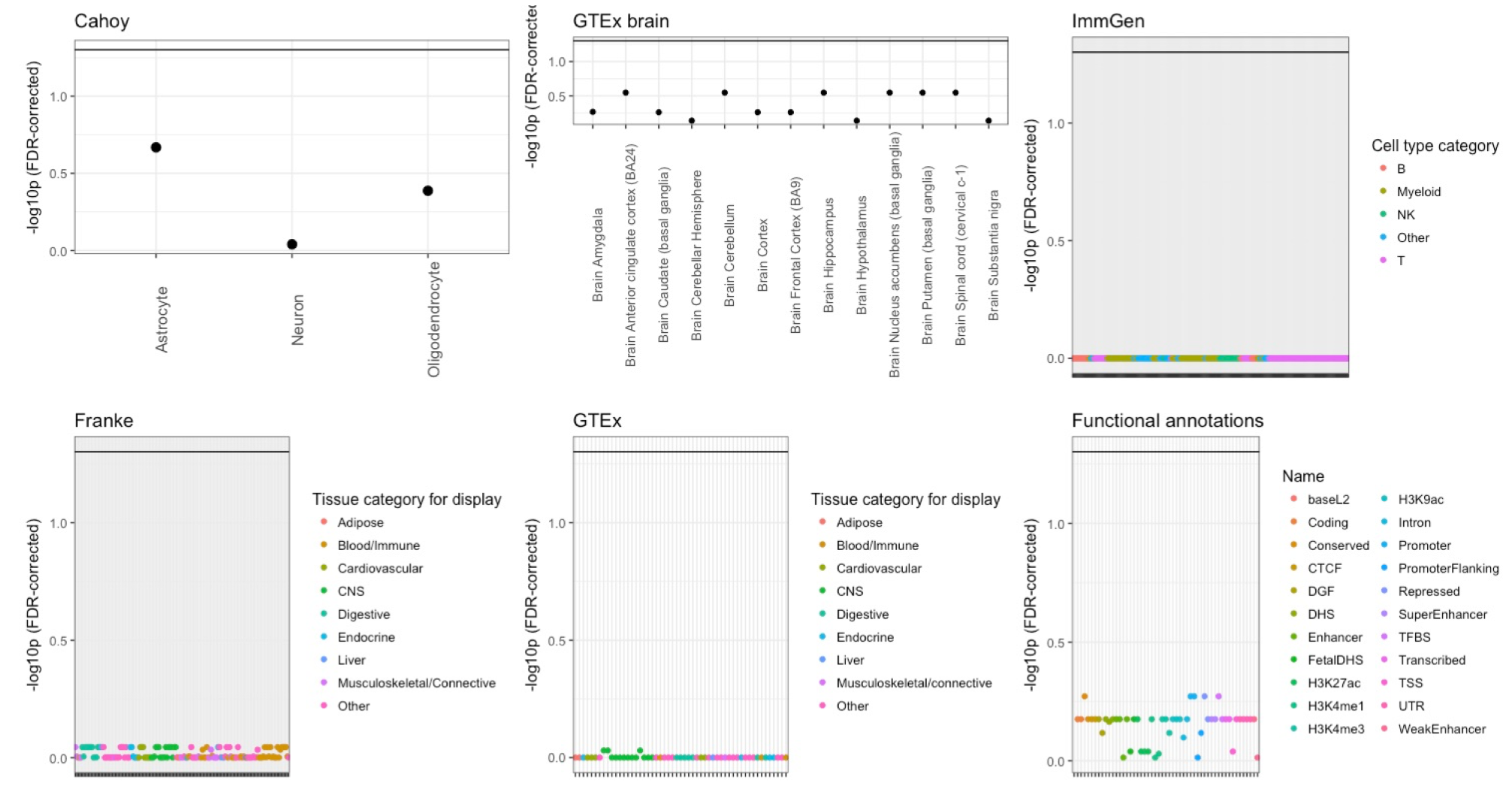

Supplement: S6 Fig — (TIF) [file pone.0227355.s006.tif]

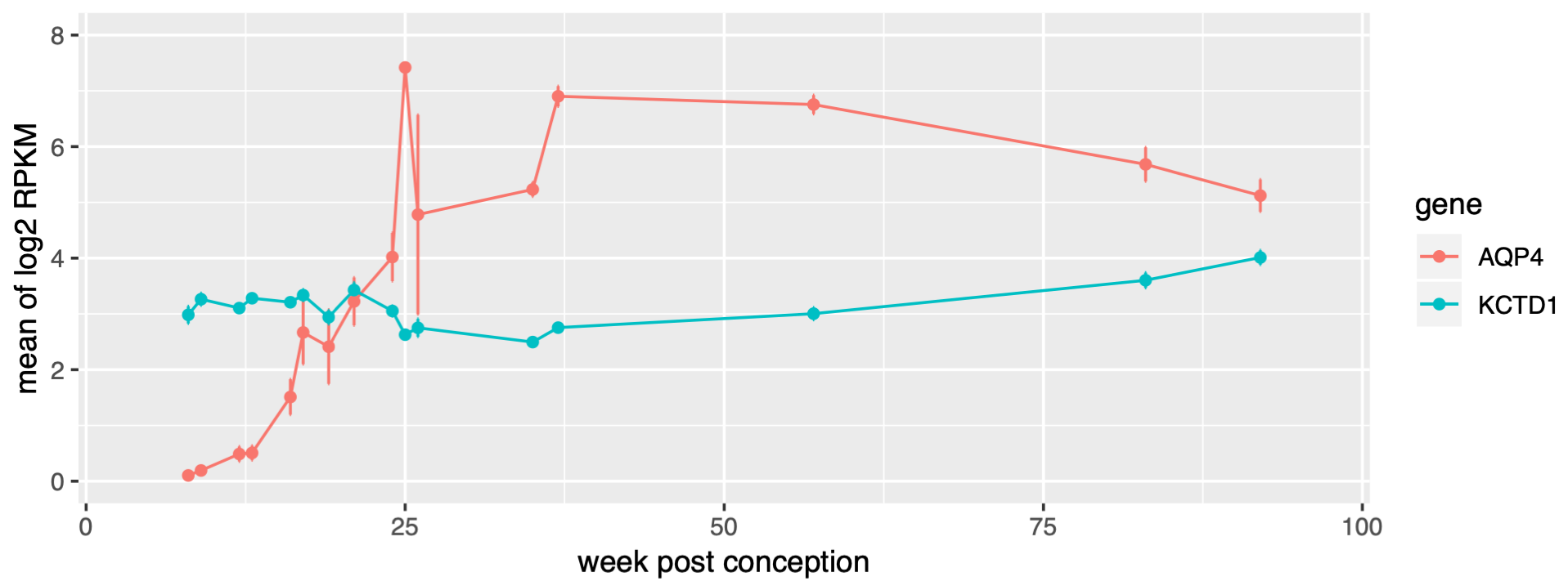

Supplement: S7 Fig — The x-axis shows the subjects’ age in weeks post conception (pcw). The y-axis depicts the mean of the log2 RPKM (reads per kilobase per million) provided by BrainSpan. AQP4 and KCTD1 are indicated by different colors. (TIF) [file pone.0227355.s007.tif]

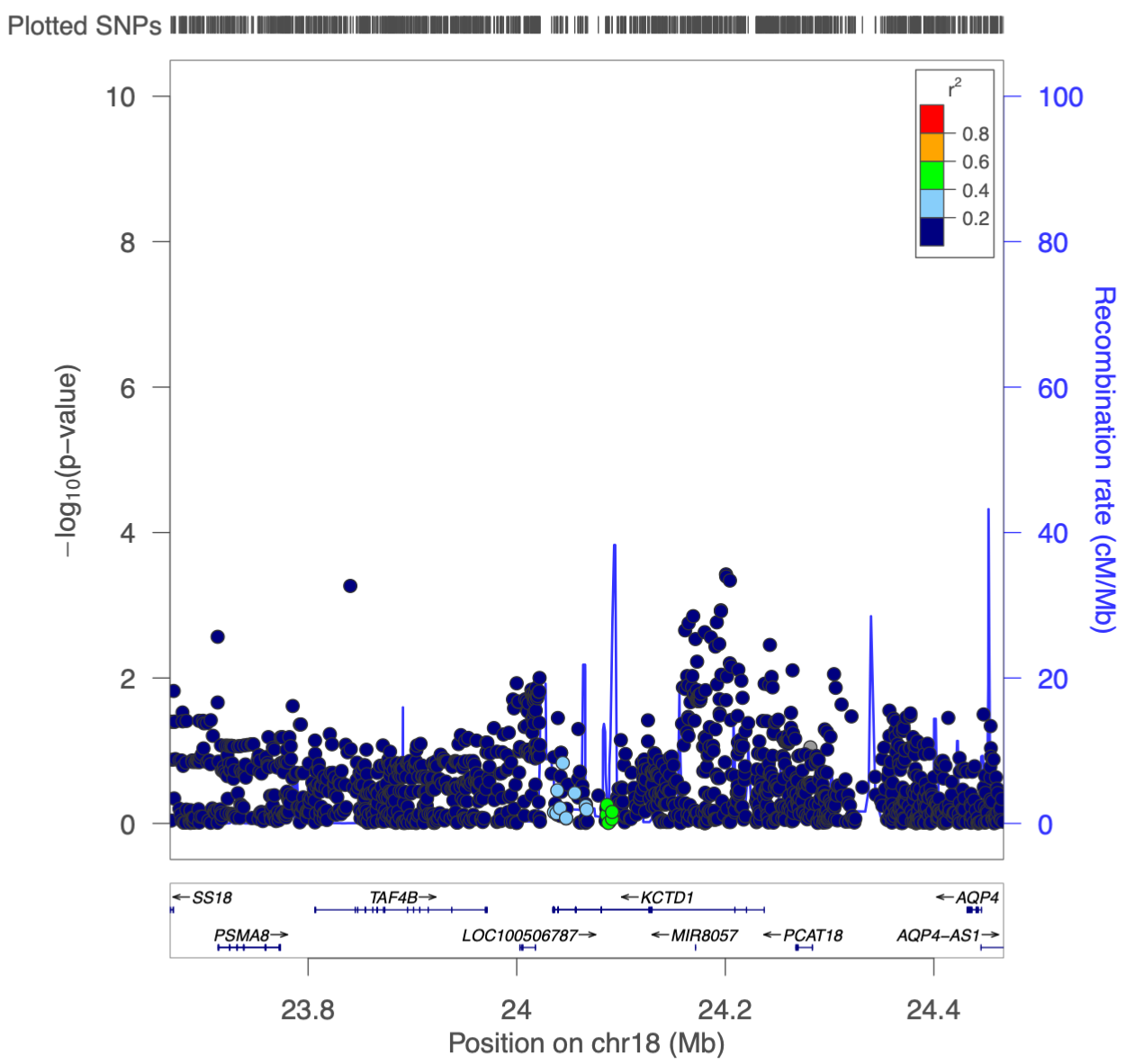

Supplement: S8 Fig — (TIF) [file pone.0227355.s008.tif]
