# Supplementary material for: Genome wide association study of incomplete hippocampal inversion in adolescents
Source: PLoS One. 2020 Jan 28;15(1):e0227355. doi: 10.1371/journal.pone.0227355 (PMC6986744; doi:10.1371/journal.pone.0227355)
Supplement: S1 File — (DOCX) [file pone.0227355.s010.docx]

IMAGEN Consortium author list:

Contact: Gunter Schumann (gunter.schumann@kcl.ac.uk)

Tobias Banaschewski^18^; Gareth J. Barker^19^; Arun L.W. Bokde^20^; Uli Bromberg^21^; Christian Büchel^21^; Erin Burke Quinlan^16^; Sylvane Desrivières^16^; Herta Flor^22,23^; Hugh Garavan^24^; Penny Gowland^25^; Bernd Ittermann^26^; Marie-Laure Paillère Martinot^27^; Eric Artiges^28^; Frauke Nees^18,22^; Dimitri Papadopoulos Orfanos^9^; Tomáš Paus^29^; Luise Poustka^30^ ; Sarah Hohmann^18^; Sabina Millenet^18^; Juliane H. Fröhner^15^; Robert Whelan^31^

^18^Department of Child and Adolescent Psychiatry and Psychotherapy, Central Institute of Mental Health, Medical Faculty Mannheim, Heidelberg University, Square J5, 68159 Mannheim, Germany; ^19^Department of Neuroimaging, Institute of Psychiatry, Psychology & Neuroscience, King’s College London, United Kingdom; ^20^Discipline of Psychiatry, School of Medicine and Trinity College Institute of Neuroscience, Trinity College Dublin; ^21^University Medical Centre Hamburg-Eppendorf, House W34, 3.OG, Martinistr. 52, 20246, Hamburg, Germany; ^22^Department of Cognitive and Clinical Neuroscience, Central Institute of Mental Health, Medical Faculty Mannheim, Heidelberg University, Square J5, Mannheim, Germany; ^23^ Department of Psychology, School of Social Sciences, University of Mannheim, 68131 Mannheim, Germany; ^24^Departments of Psychiatry and Psychology, University of Vermont, 05405 Burlington, Vermont, USA; ^25^Sir Peter Mansfield Imaging Centre School of Physics and Astronomy, University of Nottingham, University Park, Nottingham, United Kingdom; ^26^Physikalisch-Technische Bundesanstalt (PTB), Braunschweig and Berlin, Germany [or depending on journal requirements can be: Physikalisch-Technische Bundesanstalt (PTB), Abbestr. 2 – 12, Berlin, Germany; ^27^Institut National de la Santé et de la Recherche Médicale, INSERM Unit 1000 “Neuroimaging & Psychiatry”, University Paris Sud, University Paris Descartes; Sorbonne Université; and AP-HP, Department of Child and Adolescent Psychiatry, Pitié-Salpêtrière Hospital, Paris, France; ^28^Institut National de la Santé et de la Recherche Médicale, INSERM Unit 1000 “Neuroimaging & Psychiatry”, University Paris Sud, University Paris Descartes – Sorbonne Paris Cité; and Psychiatry Department 91G16, Orsay Hospital, France; ^29^Bloorview Research Institute, Holland Bloorview Kids Rehabilitation Hospital and Departments of Psychology and Psychiatry, University of Toronto, Toronto, Ontario, M6A 2E1, Canada;  ^30^Department of Child and Adolescent Psychiatry and Psychotherapy, University Medical Centre Göttingen, von-Siebold-Str. 5, 37075, Göttingen, Germany; ^31^School of Psychology and Global Brain Health Institute, Trinity College Dublin, Ireland;
